# Detecting the patient’s need for help with machine learning based on expressions

**DOI:** 10.1186/s12874-021-01502-8

**Published:** 2022-03-06

**Authors:** Lauri Lahti

**Affiliations:** Department of Computer Science, Aalto University School of Science, Espoo, Finland

**Keywords:** Personalized care, Machine learning, Convolutional neural network, Self-rating, Patient, Disabled, The need for help, Interpretation, Decision making, Coronavirus

## Abstract

**Background:**

Developing machine learning models to support health analytics requires increased understanding about statistical properties of self-rated expression statements used in health-related communication and decision making. To address this, our current research analyzes self-rated expression statements concerning the coronavirus COVID-19 epidemic and with a new methodology identifies how statistically significant differences between groups of respondents can be linked to machine learning results.

**Methods:**

A quantitative cross-sectional study gathering the “need for help” ratings for twenty health-related expression statements concerning the coronavirus epidemic on an 11-point Likert scale, and nine answers about the person’s health and wellbeing, sex and age. The study involved online respondents between 30 May and 3 August 2020 recruited from Finnish patient and disabled people’s organizations, other health-related organizations and professionals, and educational institutions (n = 673). We propose and experimentally motivate a new methodology of influence analysis concerning machine learning to be applied for evaluating how machine learning results depend on and are influenced by various properties of the data which are identified with traditional statistical methods.

**Results:**

We found statistically significant Kendall rank-correlations and high cosine similarity values between various health-related expression statement pairs concerning the “need for help” ratings and a background question pair. With tests of Wilcoxon rank-sum, Kruskal-Wallis and one-way analysis of variance (ANOVA) between groups we identified statistically significant rating differences for several health-related expression statements in respect to groupings based on the answer values of background questions, such as the ratings of suspecting to have the coronavirus infection and having it depending on the estimated health condition, quality of life and sex. Our new methodology enabled us to identify how statistically significant rating differences were linked to machine learning results thus helping to develop better human-understandable machine learning models.

**Conclusions:**

The self-rated “need for help” concerning health-related expression statements differs statistically significantly depending on the person’s background information, such as his/her estimated health condition, quality of life and sex. With our new methodology statistically significant rating differences can be linked to machine learning results thus enabling to develop better machine learning to identify, interpret and address the patient’s needs for well-personalized care.

**Supplementary Information:**

The online version contains supplementary material available at 10.1186/s12874-021-01502-8.

## Background

A self-rated health condition answered to a single question has shown a strong validity and reliability for measuring and predicting multiple dimensions of the person’s health [1, 2]. However, the self-rated health is affected by the phrasing, scales and ordering used in questions and answer options [3–6]. On the other hand, comprehensive modular questionnaire systems have been proposed and implemented, for example relying on International Classification of Functioning, Health and Disability, and Patient-Reported Outcomes Measurement Information System (PROMIS) [7, 8]. Despite the possibility to offer increasingly specifically tailored question sets and to create links between them [9, 10], a general challenge is to interpret the gained specific answers in greater agglomerated entities to make analytic conclusions and predictions in a broader context of the person’s health and wellbeing, such as in a long-term care planning and clinical decision making [11].

Furthermore, besides using predefined questionnaire structures there is a great interest for developing adaptive methods that can identify the patient’s needs from any kind of free text passages, such as from healthcare chatbots, patient diaries, online guidance and screening for care, or their derivatives, for example emergency phone calls that are immediately annotated with a speech recognition (resembling the previous proposals of [12–15]). However, according to two reviews there is still a lack of systematic development for reliable evaluation metrics for healthcare chatbots [16] and their algorithms have challenges in semantic understanding [17].

Think-aloud studies about self-rated health have identified sex- and age-dependent variations in the diversity and complexity of conceptualizations in interpretations and reasoning [5] and core categories that people use to describe and perceive health [6]. Age-related differences in self-reported opinions, attitudes or behaviors about health can also be influenced by age-induced changes in cognitive and communicative functioning [18]. There is a need to advance understandable and accurate communication between the patient and healthcare personnel and the patient’s appropriate and sufficient involvement in decision making that addresses his/her needs [19, 20].

**Fig. 1:**
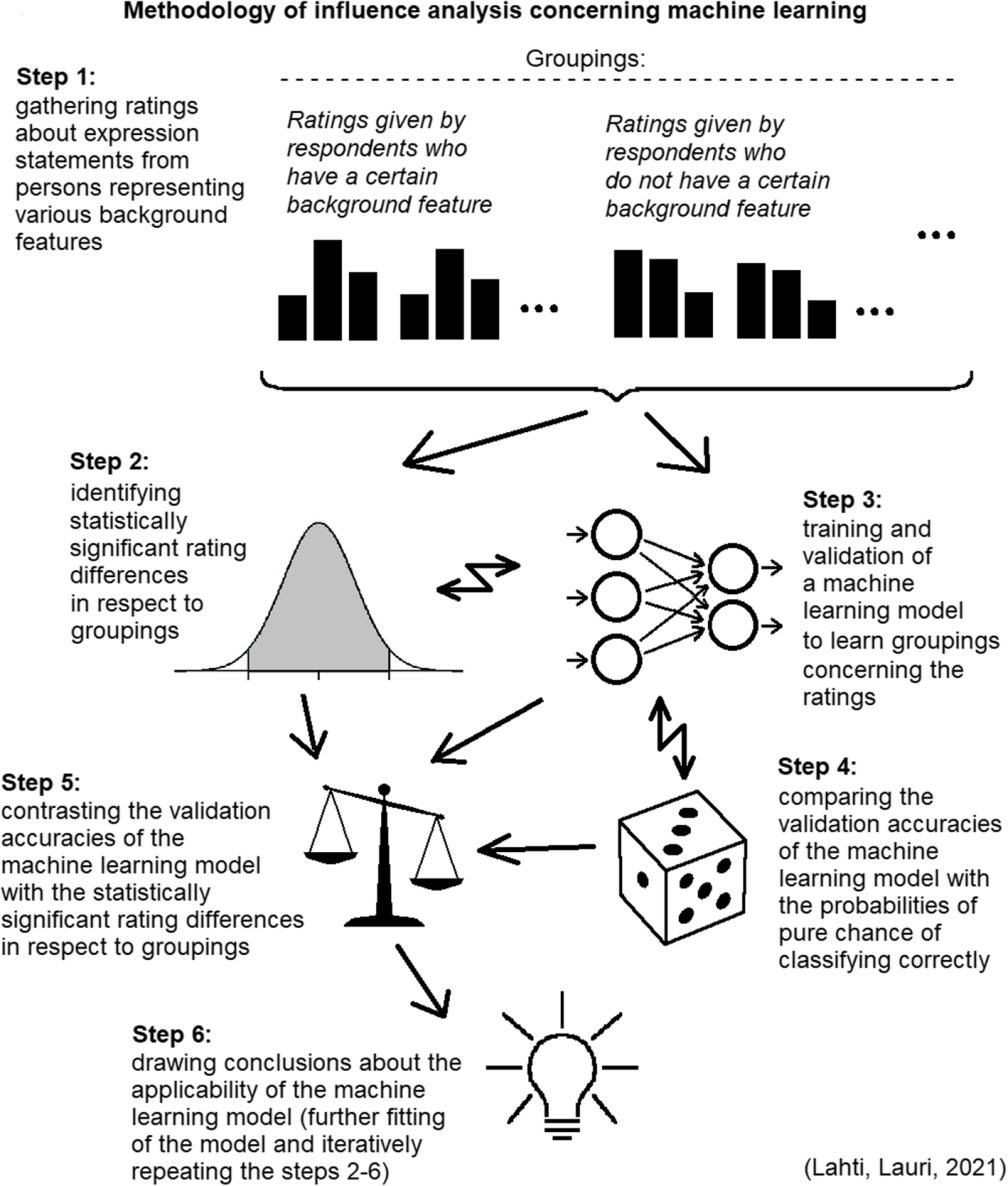
A schematic illustration about the steps 1-6 for the methodology of influence analysis concerning machine learning described in Table 1

These current challenges motivate us now to propose, develop and define a new methodology that we refer to as *influence analysis concerning machine learning*. The methodology can be used to measure the patient’s “need for help” ratings of expression statements in respect to groupings based on the answer values of background questions. Furthermore, the methodology enables to evaluate the applicability of training and validation of a machine learning model to learn the groupings concerning the ratings. The methodology enables to compare the validation accuracies of the machine learning model with the probabilities of pure chance of classifying the rating profiles correctly. In addition, the methodology enables to contrast the validation accuracies of the machine learning model with the occurrence of statistically significant rating differences for expression statements in respect to groupings based on the answer values of background questions. Table 1 summarizes the six main steps of our proposed new methodology of influence analysis concerning machine learning. Figure 1 provides a schematic illustration about the steps 1-6 for the methodology.

**Table 1:** A description of the proposed new methodology of influence analysis concerning machine learning that can be applied to measure the patient’s “need for help” ratings of expression statements in respect to groupings based on the answer values of background questions, and further to evaluate the applicability of training and validation of a machine learning model to learn the groupings concerning the ratings

*Main steps of the methodology of influence analysis concerning machine learning*
*Step 1.* Gathering questionnaire answers from persons representing various health and demographic backgrounds. - Each person gives the *“need for help” ratings* for a set of common expression statements that describe imagined scenarios. - The rating answers given by the person form his/her “need for help” rating profile. (Described in the chapter “Gathering ratings about expression statements from persons representing various background features”)
*Step 2.* Identifying statistically significant and non-significant rating differences for expression statements in respect to groupings based on the *answer values of background questions* (for example groupings relying on the person’s answer about his/her estimated health condition). (Described in the chapter “Identifying statistically significant rating differences for expression statements in respect to background questions”)
*Step 3.* Training and validation of a *machine learning model* (with a supervised learning approach) to learn the groupings concerning the “need for help” ratings. This step uses the same groupings of respondents that have been used in the step 2. (Described in the chapter “Training and validation of a machine learning model to learn groupings concerning the ratings”)
*Step 4.* Comparing the validation accuracies of the machine learning model with the probabilities of pure chance of classifying the rating profiles correctly (averaged from at least 100 separate training and validation sequences). (Described in the chapter “Comparing the validation accuracies of the machine learning model with the probabilities of pure chance”)
*Step 5.* Contrasting the validation accuracies of the machine learning model with the occurrence of statistically significant and non-significant rating differences for expression statements in respect to groupings based on the answer values of background questions (averaged from at least 100 separate training and validation sequences). (Described in the chapter “Contrasting the validation accuracies of the machine learning model with the statistically significant rating differences in respect to groupings”)
*Step 6.* Drawing conclusions about the applicability of the current machine learning model in this knowledge context. Based on the conclusions further fitting of the model and iteratively repeating the steps 2-6. (Described in the chapter “Drawing conclusions about the applicability of the current machine learning model”)

In this research article, we now focus on introducing general principles of the new methodology and describe an illustrative empirical application of the methodology with our gathered experimental data.

In accordance with the methodology presented in Table 1, the above-mentioned previous research and current challenges motivate us now to address *two main research questions (RQ)*:

- RQ1) How do different people rate the “need for help” for a set of health-related expression statements and how this rating depends on the background information about the person (such as his/her demographic information and evaluation about own health and wellbeing)? This main research question RQ1 emphasizes especially the steps 1-2 of Table 1.

- RQ2) What kinds of results can be gained when training a convolutional neural network model based on the “need for help” ratings to classify persons into groups based on their background information? This main research question RQ2 emphasizes especially the steps 3-6 of Table 1.

Relying on the methods and results developed in our previous research [21, 22], we now analyze experimental measurements (n = 673) including the “need for help” ratings for twenty health-related expression statements concerning coronavirus COVID-19 epidemic, and nine answers about the person’s health and wellbeing, sex and age. Our measuring methodology is adapted from the dimensional affective models which suggest that dimensions of pleasure, arousal, dominance and approach-avoidance have a fundamental role in human experience and response systems [23–25]. Our approach is also motivated by the previous research that has experimentally gathered a list of self-identified most significant mental imagery describing the patient’s pain combined with associated triggers, affects, meanings and avoidance patterns [26].

Resembling the previous research in the context of artificial intelligence [12–15], we wanted to evaluate the applicability of machine learning to support interpretation of the need for help in the patient’s expressions. Machine learning is a methodology that aims at learning to recognize statistical patterns in data, typically relying on either an unsupervised or supervised approach. Unsupervised learning aims at identifying naturally occurring patterns or groupings which are present in the input data and it is often challenging for humans to judge the actual appropriateness and meaningfulness of the generated groupings [11]. On the other hand, supervised learning is often carried out with an aim to predict an outcome that is based on approximating an appropriate human-made classification. Supervised learning usually tries to perform classification by choosing among subgroups such a subgroup that can best describe a new instance of data and also to produce a prediction that consists of estimating an unknown parameter [11]. Supervised learning is also actively used to estimate risk and this can be considered to extend further than just approximating the human performance and to aim at identifying hidden characteristics of data [11].

Since we aimed at identifying how the “need for help” ratings of expression statements can be used to classify persons into groups based on their background information, it was natural for us to focus now on experimenting with the supervised learning approach. To implement supervised learning, various alternative types of functions can be chosen to relate predicted values to the features that are present in the data, and these functions typically offer more flexibility for modeling than for example logistic regression models of traditional statistics [11]. These functions can be based on various alternative machine learning models, and among them artificial neural networks have achieved a high accuracy in classification tasks [13]. Models relying on artificial neural networks with multiple layers represent an approach often referred to as deep learning [13]. Relying on a literature review and some initial comparative experimenting with popular and openly available models we decided to use a convolutional neural network model in our machine learning experiments since it has been successfully applied in classification of medical literature, patient records, clinical narratives and patient phenotypes [12–15, 27–29], and it achieves good results with both image and textual input data [30].

In respect to the coronavirus COVID-19 epidemic, artificial neural networks have been applied to classify coronavirus-related online discussions and then to supply them with an emotional labeling based on a pre-existing emotion vocabulary and rules [31].

## Methods

### 1. Gathering ratings about expression statements from persons representing various background features

In accordance with Table 1, our proposed new methodology in respect to the step 1 consists of gathering questionnaire answers from persons representing various health and demographic backgrounds.

### 1.1 Study design, setting, participants and sampling strategy

We carried out a quantitative cross-sectional study that gathered online questionnaire answers from 673 unique persons that we recruited consecutively from various Finnish patient and disabled people’s organizations, other health and wellness organizations, and educational institutions as well as organizations of healthcare professionals in the time period ranging from 30 May to 3 August 2020 based on a consecutive sampling approach. When accessing the online questionnaire at the Finnish web server of our DIHEML research project (https://ilmaisu.cs.aalto.fi/research/welcome), the person was informed that only persons who are at least 16 years old are allowed to participate. Furthermore, to address the General Data Protection Regulation of the European Union a privacy notice about the research was shown to the person and he/she was asked to give an approval for handling his/her data.

#### 1.2 Variables and study size

 Based on the earlier health studies [32] a suitable sample size was identified for analyzing how the “need for help” ratings about expression statements depend on the background information about the person (addressing the main research question RQ1) and analyzing the validity of the machine learning method and its comparison to traditional statistical methods (addressing the main research question RQ2). We gathered twenty rating answers that measured the degree of the “need for help” that the person associated with the imagined care situations related to the coronavirus COVID-19 epidemic. In addition, we gathered nine answers about the person’s background information. All these answers were gathered as a part of a greater data acquisition entity [33, 34] for our research that aims at development of a care decision-making model, with some supplementing questionnaire items that will be reported in a more detail in another future publication.

#### 1.3 Data sources/measurement

 We gathered online questionnaire answers so the that the person gave each answer by selecting one of the available alternative answer options, as shown in Tables 2 and 3. We publish an anonymized version of our current research data (the open access data set “Need for help related to the coronavirus COVID-19 epidemic”) in the supplementing spreadsheet file Additional file 2. We also publish additional details about our research methodology, measurements and analysis results in the supplementing document Data analysis supplement (Additional file 1).

**Table 2:** Expression statements (ES) concerning the coronavirus COVID-19 epidemic that were rated by the person in respect to the impression about the “need for help”

*Compact notation*	*Expression statement*	*Range of values for the person’s answer (indicating the “need for help” rating)*
ES1	“I have a flu.”	0-10
ES2	“I have a cough.”	0-10
ES3	“I have a shortness of breath.”	0-10
ES4	“My health condition is weakening.”	0-10
ES5	“I have a sore throat.”	0-10
ES6	“I have muscular ache.”	0-10
ES7	“I have a fever.”	0-10
ES8	“A sudden fever rises for me with 38 degrees of Celsius or more.”	0-10
ES9	“I suspect that I have now become infected by the coronavirus.”	0-10
ES10	“I have now become infected by the coronavirus.”	0-10
ES11	“I am quarantined from meeting other people ordinarily so that the spreading of an infectious disease could be prevented.”	0-10
ES12	“I must be inside a house without getting out.”	0-10
ES13	“I must be without a human companion.”	0-10
ES14	“I do not cope in everyday life independently without getting help from other persons.”	0-10
ES15	“I do not cope at home independently without getting help from persons who originate outside of my home.”	0-10
ES16	“I have an infectious disease.”	0-10
ES17	“I have an infectious disease that has been verified by a doctor.”	0-10
ES18	“I suspect that I have an infectious disease.”	0-10
ES19	“I have a bad health condition.”	0-10
ES20	“I have an ordinary health condition.”	0-10

**Table 3:** Background questions (BQ) presented to the person

*Compact notation*	*Question about the person’s background information*	*Range of values for the person’s answer*
BQ1: an estimated health condition	“What kind of health condition you have currently according to your opinion?” [32, 35]	A 9-point Likert scale supplied with the following partial labeling: “9 Good”, “8 –“, “7 Rather good”, “6 –“, “5 Medium”, “4 –“, “3 Rather bad”, “2 –“, “1 Bad”.
BQ2: a health problem reduces ability	“Do you have a permanent or long-lasting disease or such deficit, ailment or disability that reduces your ability to work or to perform your daily living activities? Here the question refers to all long-lasting diseases identified by a doctor, and also to such ailments not identified by a doctor which have lasted at least three months but which affect your ability to perform your daily living activities.“ [32]	No or yes.
BQ3: one or more diseases identified by a doctor	“Has there been a situation that a doctor has identified in you one or several of the following diseases?“ [32]	The person answers by selecting one or more options from a list of diseases [18], see details in Data analysis supplement (Additional file 1). For some options there is a question “other, what?” and an adjacent text input box so that the person can write some additional information concerning that option.
BQ4: a continuous or repeated need for a doctor’s care	“Do you need continuously or repeatedly care given by a doctor for a long-lasting disease, deficit or disability that you have just mentioned?“ [32]	No or yes.
BQ5: the quality of life	“How would you rate your quality of life? Give your estimate based on the latest two weeks.” [36, 37]	A 9-point Likert scale supplied with the following partial labeling: “9 Very good”, “8 –“, “7 Good”, “6 –“, “5 Neither good nor bad”, “4 –“, “3 Bad”, “2 –“, “1 Very bad”.
BQ6: the satisfaction about health	“How satisfied are you with your health? Give your estimate based on the latest two weeks.” [36, 37]	A 9-point Likert scale supplied with the following partial labeling: “9 Very satisfied”, “8 –“, “7 Satisfied”, “6 –“, “5 Neither satisfied nor dissatisfied”, “4 –“, “3 Dissatisfied”, “2 –“, “1 Very dissatisfied”.
BQ7: the satisfaction about ability	“How satisfied are you with your ability to perform your daily living activities? Give your estimate based on the latest two weeks.” [36, 37]	A 9-point Likert scale supplied with the following partial labeling: “9 Very satisfied”, “8 –“, “7 Satisfied”, “6 –“, “5 Neither satisfied nor dissatisfied”, “4 –“, “3 Dissatisfied”, “2 –“, “1 Very dissatisfied”.
BQ8: the sex	“Tell what is your sex. The answer alternatives are similar as in the earlier health surveys of Finnish Institute for Health and Welfare (THL) to maintain comparability with the earlier results.” [32]	Man or woman.
BQ9: the age	“Tell what is your age.” [32]	Age in years selected from the following range: 16 years, 17 years, …, 99 years, 100 years or more.

### 1.4 Bias

 As motivated in the chapters “Methods” and “Results”, due to the overall complexity of modeling semantics of a natural language and the limited size of the current data set our gained results are not meant to introduce a model that can actually learn the groupings very well. Instead, we aim now to propose and experimentally motivate a new methodology that can be used for analyzing how the machine learning models are influenced by the properties of the data so that these notions can be exploited to develop better machine learning models.

### 1.5 Quantitative variables and statistical methods

 To simplify practical calculations in the data analysis, the original “need for help” rating answer values in the range 0-10 were transformed linearly to a new range 0.0-1.0. To address our main research question RQ1, we use traditional statistical tests to evaluate overall answer distributions. We computed Kendall rank-correlation and cosine similarity measures for each comparable pair of parameter values of the “need for help” ratings of expression statements ES1-ES20 and the answers of the background questions BQ1 and BQ5-BQ7. Motivated by a recommendation of [38] we considered a Kendall rank-correlation measure greater than or equal to 0.70 to indicate a significant correlation and the statistical significance levels were defined as p < 0.05, p < 0.01 and p < 0.001. Before computing cosine similarity measures the answer values of each parameter were normalized by the formula *(x - min(x))/(max(x)-min(x))* and then these new values were shifted so that the mean value was positioned to the zero by the formula *(x - mean(x))*.

We computed Wilcoxon rank-sum test (i.e., Mann–Whitney U test) between two groups and Kruskal-Wallis test between three groups to identify statistically significant rating differences for each expression statement in respect to groupings based on the answer values of each background question (groupings are shown in Table 4). In respect to the background questions BQ1-BQ2 and BQ4-BQ8 we created groupings of two groups so that the “group 1” contained those respondents who gave an answer value that was lower than the mean value of all the answer values to the background question, and the “group 2” contained all the other respondents. In respect to the background question BQ8 (the age) we created groupings of two groups so that the “group 1” contained those respondents who gave an answer value that was lower than the median value of all the answer values to the background question, and the “group 2” contained all the other respondents. We created groupings of three groups so that the respondents could be divided the most evenly into three ranges of answer values of the background question. The statistical significance levels were defined as p < 0.05, p < 0.01 and p < 0.001. We computed supplementing tests of one-way analysis of variance (ANOVA) between two groups and between three groups to identify statistically significant rating differences for the same expression statements as Wilcoxon rank-sum test and Kruskal-Wallis test.

**Table 4:** Expression statements (ES) having statistically significant the “need for help” rating differences in the grouping based on the answer values of each background question (BQ), and evaluation about how well the convolutional neural network model can learn a labeling that matches the grouping (n = 673). M = mean, Mdn=median, SD=standard deviation

	*Statistically significant rating differences*	*Training and validation metrics*^*2*^ *of the convolutional neural network model to learn a labeling that matches the grouping*					*Comparison of the validation accuracy with the probability of pure chance*	
*Grouping based on the answer value (x) of the background question (BQ)*	*Expression statements (ES) having statistically significant rating differences in the grouping (the difference of mean ratings*^*1*^ *about the need for help)*	*Epoch step*	*Training loss*	*Training accuracy*	*Validation loss*	*Validation accuracy*	*Probability of pure chance of classifying the rating profiles correctly*^*3*^ *(based on the size of the greatest group)*	*Difference of the mean validation accuracy and the probability of pure chance of classifying the rating profiles correctly*
BQ1, two groups: x < 7 (n_1_=263), x>=7 (n_2_=410)	ES6: diff_g1&g2_=0,07 (95% CI [0,03; 0,11], p = 0.0011); ES8: diff_g1&g2_=-0,08 (95% CI [-0,14; -0,02], p = 0.0073); ES9: diff_g1&g2_=-0,08 (95% CI [-0,14; -0,02], p = 0.0068); ES10: diff_g1&g2_=-0,09 (95% CI [-0,15; -0,02], p = 0.0049); ES7: diff_g1&g2_=-0,05 (95% CI [-0,10; 0,00], p = 0.0384); ES16: diff_g1&g2_=-0,06 (95% CI [-0,12; 0,00], p = 0.0403); ES17: diff_g1&g2_=-0,08 (95% CI [-0,14; -0,02], p = 0.0143); ES18: diff_g1&g2_=-0,05 (95% CI [-0,10; 0,00], p = 0.0358);	M = 11.26 Mdn=11 SD=2.39	M = 0.55 Mdn=0.55 SD=0.03	M = 0.73 Mdn=0.72 SD=0.02	M = 0.59 Mdn=0.59 SD=0.01	M = 0.69 Mdn=0.69 SD=0.02	0.61	0.08
BQ1, three groups: x < 6 (n_1_= 218), 6<=x < 8 (n_2_=207), x>=8 (n_3_=248)	ES5: diff_g1&g3_=-0,01 (95% CI [-0,05; 0,03], p = 0.446), diff_g1&g2_=-0,05 (95% CI [-0,09; 0,00], p = 0.054), diff_g2&g3_=0,04 (95% CI [0,00; 0,08], p = 0.102), p_g1&g2&g3_=0.0449; ES8: diff_g1&g3_=-0,08 (95% CI [-0,15; -0,02], p = 0.067), diff_g1&g2_=-0,07 (95% CI [-0,14; 0,00], p = 0.067), diff_g2&g3_=-0,01 (95% CI [-0,07; 0,06], p = 0.929), p_g1&g2&g3_=0.0489; ES9: diff_g1&g3_=-0,09 (95% CI [-0,16; -0,02], p = 0.050), diff_g1&g2_=-0,08 (95% CI [-0,15; 0,00], p = 0.058), diff_g2&g3_=-0,01 (95% CI [-0,08; 0,06], p = 0.891), p_g1&g2&g3_=0.0355; ES11: diff_g1&g3_=0,08 (95% CI [0,02; 0,14], p = 0.015), diff_g1&g2_=0,01 (95% CI [-0,05; 0,07], p = 0.858), diff_g2&g3_=0,07 (95% CI [0,02; 0,13], p = 0.015), p_g1&g2&g3_=0.0108;	M = 4.85 Mdn=4 SD=1.8	M = 1.02 Mdn=1.03 SD=0.03	M = 0.48 Mdn=0.47 SD=0.03	M = 1.06 Mdn=1.06 SD=0.01	M = 0.40 Mdn=0.40 SD=0.02	0.37	0.03
BQ2, two groups: x < 2 (n_1_=219), x>=2 (n_2_=454)	ES11: diff_g1g2_=-0,08 (95% CI [-0,13; -0,03], p = 0.0014); ES6: diff_g1g2_=-0,06 (95% CI [-0,10; -0,02], p = 0.0039); ES3: diff_g1g2_=0,06 (95% CI [0,01; 0,12], p = 0.0476); ES14: diff_g1g2_=0,06 (95% CI [-0,01; 0,12], p = 0.04); ES15: diff_g1g2_=0,08 (95% CI [0,01; 0,14], p = 0.0189);	M = 5.55 Mdn=6 SD=2.91	M = 0.57 Mdn=0.56 SD=0.04	M = 0.69 Mdn=0.69 SD=0.02	M = 0.63 Mdn=0.63 SD=0.01	M = 0.66 Mdn=0.66 SD=0.02	0.67	-0.01
BQ4, two groups: x < 2 (n_1_=364), x>=2 (n_2_=309)	ES6: diff_g1g2_=-0,06 (95% CI [-0,09; -0,02], p = 0.0064); ES11: diff_g1g2_=-0,06 (95% CI [-0,10; -0,01], p = 0.0165);	M = 3.44 Mdn=3 SD=1.28	M = 0.67 Mdn=0.67 SD=0.01	M = 0.59 Mdn=0.60 SD=0.02	M = 0.68 Mdn=0.67 SD=0	M = 0.57 Mdn=0.57 SD=0.02	0.54	0.03
BQ5, two groups: x < 7 (n_1_=274), x>=7 (n_2_=399)	ES6: diff_g1g2_=0,06 (95% CI [0,02; 0,10], p = 0.0024); ES9: diff_g1g2_=-0,08 (95% CI [-0,14; -0,02], p = 0.0043); ES10: diff_g1g2_=-0,08 (95% CI [-0,15; -0,02], p = 0.0036); ES11: diff_g1g2_=0,06 (95% CI [0,01; 0,11], p = 0.0168); ES16: diff_g1g2_=-0,06 (95% CI [-0,12; -0,01], p = 0.0271); ES17: diff_g1g2_=-0,07 (95% CI [-0,13; -0,01], p = 0.0303);	M = 3.27 Mdn=3 SD=1.65	M = 0.64 Mdn=0.63 SD=0.02	M = 0.65 Mdn=0.65 SD=0.03	M = 0.66 Mdn=0.67 SD=0	M = 0.60 Mdn=0.60 SD=0.02	0.59	0,01
BQ5, three groups: x < 6 (n_1_=190), 6<=x < 8 (n_2_=271), x>=8 (n_3_=212)	ES9: diff_g1&g3_=-0,15 (95% CI [-0,22; -0,07], p = 0.0005), diff_g1&g2_=-0,09 (95% CI [-0,17; -0,02], p = 0.0230), diff_g2&g3_=-0,05 (95% CI [-0,12; 0,02], p = 0.0965), p_g1&g2&g3_=0.0007; ES10: diff_g1&g3_=-0,15 (95% CI [-0,23; -0,07], p = 0.0004), diff_g1&g2_=-0,09 (95% CI [-0,17; -0,02], p = 0.0112), diff_g2&g3_=-0,06 (95% CI [-0,13; 0,02], p = 0.1699), p_g1&g2&g3_=0.0005; ES6: diff_g1&g3_=0,07 (95% CI [0,03; 0,12], p = 0.016), diff_g1&g2_=0,02 (95% CI [-0,02; 0,07], p = 0.393), diff_g2&g3_=0,05 (95% CI [0,01; 0,09], p = 0.023), p_g1&g2&g3_=0.0093; ES8: diff_g1&g3_=-0,11 (95% CI [-0,18; -0,04], p = 0.013), diff_g1&g2_=-0,09 (95% CI [-0,16; -0,02], p = 0.013), diff_g2&g3_=-0,02 (95% CI [-0,08; 0,05], p = 0.985), p_g1&g2&g3_=0.0117; ES16: diff_g1&g3_=-0,09 (95% CI [-0,16; -0,02], p = 0.04), diff_g1&g2_=-0,08 (95% CI [-0,15; -0,01], p = 0.04), diff_g2&g3_=-0,01 (95% CI [-0,08; 0,05], p = 0.76), p_g1&g2&g3_=0.0301; ES17: diff_g1&g3_=-0,10 (95% CI [-0,18; -0,03], p = 0.04), diff_g1&g2_=-0,08 (95% CI [-0,15; -0,01], p = 0.06), diff_g2&g3_=-0,02 (95% CI [-0,09; 0,04], p = 0.53), p_g1&g2&g3_=0.0329; ES20: diff_g1&g3_=0,04 (95% CI [-0,02; 0,09], p = 0.022), diff_g1&g2_=-0,01 (95% CI [-0,07; 0,04], p = 0.928), diff_g2&g3_=0,05 (95% CI [0,00; 0,10], p = 0.022), p_g1&g2&g3_=0.0139;	M = 3.63 Mdn=4 SD=1.33	M = 1.05 Mdn=1.05 SD=0.02	M = 0.44 Mdn=0.44 SD=0.03	M = 1.07 Mdn=1.07 SD=0.01	M = 0.42 Mdn=0.43 SD=0.03	0.40	0.02
BQ6, two groups: x < 7 (n_1_=318), x>=7 (n_2_=355)	ES11: diff_g1&g2_=0,08 (95% CI [0,04; 0,13], p = 0.0006); ES6: diff_g1&g2_=0,06 (95% CI [0,02; 0,09], p = 0.0056);	M = 5.35 Mdn=5 SD=1.61	M = 0.62 Mdn=0.63 SD=0.02	M = 0.63 Mdn=0.63 SD=0.03	M = 0.65 Mdn=0.65 SD=0	M = 0.60 Mdn=0.60 SD=0,02	0.53	0.07
BQ6, three groups: x < 6 (n_1_=240), 6<=x < 8 (n_2_=229), x>=8 (n_3_=204)	ES11: diff_g1&g3_=0,09 (95% CI [0,03; 0,15], p = 0.0077), diff_g1&g2_=0,03 (95% CI [-0,03; 0,08], p = 0.3516), diff_g2&g3_=0,06 (95% CI [0,00; 0,12], p = 0.0649), p_g1&g2&g3_=0.0098; ES6: diff_g1&g3_=0,07 (95% CI [0,02; 0,12], p = 0.019), diff_g1&g2_=0,04 (95% CI [0,00; 0,09], p = 0.141), diff_g2&g3_=0,03 (95% CI [-0,02; 0,07], p = 0.141), p_g1&g2&g3_=0.0199;	M = 3.89 Mdn=4 SD=1.8	M = 1.05 Mdn=1.06 SD=0.03	M = 0.41 Mdn=0.41 SD=0.03	M = 1.08 Mdn=1.08 SD=0	M = 0.39 Mdn=0.39 SD=0.03	0.36	0.03
BQ7, two groups: x < 7 (n_1_=201), x>=7 (n_2_=472)	ES6: diff_g1&g2_=0,08 (95% CI [0,04; 0,12], p = 0.0005); ES11: diff_g1&g2_=0,07 (95% CI [0,02; 0,12], p = 0.0078); ES19: diff_g1&g2_=0,07 (95% CI [0,02; 0,11], p = 0.0048);	M = 7.26 Mdn=7 SD=1.63	M = 0.53 Mdn=0.54 SD=0.02	M = 0.75 Mdn=0.74 SD=0.01	M = 0.59 Mdn=0.59 SD=0	M = 0.72 Mdn=0.72 SD=0.01	0.70	0.02
BQ7, three groups: x < 6 (n_1_=143), 6<=x < 8 (n_2_=214), x>=8 (n_3_=316)	ES6: diff_g1&g3_=0,09 (95% CI [0,04; 0,13], p = 0.0051), diff_g1&g2_=0,04 (95% CI [-0,01; 0,09], p = 0.1801), diff_g2&g3_=0,05 (95% CI [0,00; 0,09], p = 0.0619), p_g1&g2&g3_=0.0042; ES11: diff_g1&g3_=0,10 (95% CI [0,04; 0,16], p = 0.0086), diff_g1&g2_=0,03 (95% CI [-0,04; 0,09], p = 0.4526), diff_g2&g3_=0,07 (95% CI [0,02; 0,12], p = 0.0186), p_g1&g2&g3_=0.0035;	M = 1.31 Mdn=1 SD=0.61	M = 1.05 Mdn=1.06 SD=0.02	M = 0.45 Mdn=0.45 SD=0.02	M = 1.07 Mdn=1.07 SD=0.01	M = 0.47 Mdn=0.48 SD=0.02	0.47	0.00
BQ8, two groups: x < 2 (n_1_=123), x>=2 (n_2_=550)	ES4: diff_g1&g2_=-0,11 (95% CI [-0,17; -0,05], p = 0.0001); ES12: diff_g1&g2_=-0,13 (95% CI [-0,20; -0,06], p = 0.0002); ES14: diff_g1&g2_=-0,20 (95% CI [-0,27; -0,13], p = 0.0000); ES15: diff_g1&g2_=-0,20 (95% CI [-0,28; -0,12], p = 0.0000); ES3: diff_g1&g2_=-0,10 (95% CI [-0,16; -0,03], p = 0.0031); ES10: diff_g1&g2_=-0,12 (95% CI [-0,20; -0,04], p = 0.0050); ES11: diff_g1&g2_=-0,08 (95% CI [-0,14; -0,02], p = 0.0058); ES8: diff_g1&g2_=-0,09 (95% CI [-0,16; -0,02], p = 0.0225); ES9: diff_g1&g2_=-0,10 (95% CI [-0,18; -0,03], p = 0.0142); ES13: diff_g1&g2_=-0,07 (95% CI [-0,14; -0,01], p = 0.0223); ES16: diff_g1&g2_=-0,10 (95% CI [-0,17; -0,02], p = 0.0159); ES17: diff_g1&g2_=-0,09 (95% CI [-0,17; -0,02], p = 0.0319); ES18: diff_g1&g2_=-0,08 (95% CI [-0,14; -0,01], p = 0.0242);	M = 6.14 Mdn=6 SD=1.69	M = 0.42 Mdn=0.42 SD=0.02	M = 0.83 Mdn=0.83 SD=0.01	M = 0.48 Mdn=0.48 SD=0.01	M = 0.79 Mdn=0.78 SD=0.01	0.82	-0.03
BQ9, two groups: x < 51 (n_1_=333), x>=51 (n_2_=340)	ES1: diff_g1&g2_=0,09 (95% CI [0,05; 0,12], p = 0.0000); ES2: diff_g1&g2_=0,10 (95% CI [0,06; 0,13], p = 0.0000); ES3: diff_g1&g2_=0,13 (95% CI [0,08; 0,18], p = 0.0000); ES4: diff_g1&g2_=0,10 (95% CI [0,05; 0,15], p = 0.0006); ES5: diff_g1&g2_=0,08 (95% CI [0,04; 0,11], p = 0.0000); ES14: diff_g1&g2_=0,14 (95% CI [0,08; 0,19], p = 0.0001); ES15: diff_g1&g2_=0,14 (95% CI [0,08; 0,20], p = 0.0001); ES7: diff_g1&g2_=0,06 (95% CI [0,01; 0,10], p = 0.0133); ES8: diff_g1&g2_=0,09 (95% CI [0,04; 0,15], p = 0.0466); ES11: diff_g1&g2_=-0,05 (95% CI [-0,09; 0,00], p = 0.0485); ES13: diff_g1&g2_=0,05 (95% CI [0,01; 0,10], p = 0.0297); ES19: diff_g1&g2_=0,04 (95% CI [0,00; 0,08], p = 0.0193);	M = 5.79 Mdn=6 SD=1.44	M = 0.58 Mdn=0.58 SD=0.02	M = 0.69 Mdn=0.69 SD=0.02	M = 0.61 Mdn=0.61 SD=0.01	M = 0.68 Mdn=0.68 SD=0.02	0.51	0.17
BQ9, three groups: x < 40 (n_1_=225), 40<=x < 60 (n_2_=231), x>=60 (n_3_=217)	ES1: diff_g1&g3_=0,10 (95% CI [0,06; 0,14], p = 0.0000), diff_g1&g2_=0,07 (95% CI [0,03; 0,11], p = 0.0002), diff_g2&g3_=0,03 (95% CI [-0,01; 0,06], p = 0.0716), p_g1&g2&g3_=0.0000; ES2: diff_g1&g3_=0,12 (95% CI [0,08; 0,16], p = 0.0000), diff_g1&g2_=0,07 (95% CI [0,03; 0,11], p = 0.0007), diff_g2&g3_=0,05 (95% CI [0,01; 0,09], p = 0.0162), p_g1&g2&g3_=0.0000; ES3: diff_g1&g3_=0,17 (95% CI [0,11; 0,23], p = 0.0000), diff_g1&g2_=0,06 (95% CI [0,01; 0,12], p = 0.110), diff_g2&g3_=0,10 (95% CI [0,04; 0,17], p = 0.003), p_g1&g2&g3_=0.0000; ES4: diff_g1&g3_=0,13 (95% CI [0,07; 0,19], p = 0.0011), diff_g1&g2_=0,02 (95% CI [-0,04; 0,07], p = 0.5450), diff_g2&g3_=0,11 (95% CI [0,05; 0,18], p = 0.0011), p_g1&g2&g3_=0.0004; ES5: diff_g1&g3_=0,09 (95% CI [0,04; 0,13], p = 0.0000), diff_g1&g2_=0,03 (95% CI [-0,01; 0,08], p = 0.042), diff_g2&g3_=0,05 (95% CI [0,01; 0,10], p = 0.012), p_g1&g2&g3_=0.0000; ES14: diff_g1&g3_=0,17 (95% CI [0,11; 0,24], p = 0.0002), diff_g1&g2_=0,05 (95% CI [-0,01; 0,12], p = 0.6097), diff_g2&g3_=0,12 (95% CI [0,05; 0,19], p = 0.0031), p_g1&g2&g3_=0.0002; ES15: diff_g1&g3_=0,18 (95% CI [0,11; 0,25], p = 0.0004), diff_g1&g2_=0,06 (95% CI [0,00; 0,13], p = 0.5430), diff_g2&g3_=0,12 (95% CI [0,04; 0,19], p = 0.0082), p_g1&g2&g3_=0.0006; ES7: diff_g1&g3_=0,09 (95% CI [0,03; 0,15], p = 0.0064), diff_g1&g2_=0,01 (95% CI [-0,04; 0,06], p = 0.7293), diff_g2&g3_=0,08 (95% CI [0,02; 0,14], p = 0.0120), p_g1&g2&g3_=0.0043; ES11: diff_g1&g3_=-0,08 (95% CI [-0,14; -0,03], p = 0.0069), diff_g1&g2_=-0,10 (95% CI [-0,15; -0,04], p = 0.0033), diff_g2&g3_=0,02 (95% CI [-0,04; 0,07], p = 0.5752), p_g1&g2&g3_=0.0017; ES8: diff_g1&g3_=0,13 (95% CI [0,06; 0,20], p = 0.016), diff_g1&g2_=0,03 (95% CI [-0,04; 0,09], p = 0.956), diff_g2&g3_=0,11 (95% CI [0,04; 0,18], p = 0.016), p_g1&g2&g3_=0.0116; ES10: diff_g1&g3_=0,14 (95% CI [0,07; 0,22], p = 0.034), diff_g1&g2_=0,02 (95% CI [-0,05; 0,09], p = 0.995), diff_g2&g3_=0,12 (95% CI [0,04; 0,20], p = 0.034), p_g1&g2&g3_=0.0245; ES19: diff_g1&g3_=0,06 (95% CI [0,01; 0,11], p = 0.034), diff_g1&g2_=0,03 (95% CI [-0,01; 0,08], p = 0.198), diff_g2&g3_=0,03 (95% CI [-0,03; 0,08], p = 0.198), p_g1&g2&g3_=0.0351; ES20: diff_g1&g3_=-0,07 (95% CI [-0,12; -0,02], p = 0.127), diff_g1&g2_=0,01 (95% CI [-0,04; 0,06], p = 0.268), diff_g2&g3_=-0,08 (95% CI [-0,13; -0,02], p = 0.026), p_g1&g2&g3_=0.0253;	M = 7.13 Mdn=7 SD=1.45	M = 0.93 Mdn=0.93 SD=0.03	M = 0.54 Mdn=0.54 SD=0.03	M = 0.98 Mdn=0.98 SD=0.01	M = 0.50 Mdn=0.50 SD=0.03	0.34	0.16

^1^ For groupings of two groups the difference of mean ratings (each mean rating in the range 0.0-1.0) is computed by the formula *(M*_*1*_*-M*_*2*_*)*, and for groupings of three groups by the formula *max({(M*_*1*_*-M*_*3*_*),(M*_*1*_*-M*_*2*_*),(M*_*2*_*-M*_*3*_*)})*. Wilcoxon rank-sum test (for two groups) and Kruskal-Wallis test (for three groups) indicate the statistically significant rating differences (p < 0.05) between groups, each rating difference (*diff*_*g1&g3*_, *diff*_*g1&g2*_ and *diff*_*g2&g3*_) supplied with the corresponding 95% confidence interval (CI) and the p-value of the Wilcoxon pairwise comparison. The parameter *p*_*g1&g2&g3*_ shows the p-value of the Kruskal-Wallis test for three groups

^2^ Training and validation metrics of the convolutional neural network model are averaged from 100 separate training and validation sequences to learn a labeling that matches the grouping (n = 673)

^3^ For groupings of two groups the probability of pure chance of classifying the rating profiles correctly is computed by the formula *(max({n*_*1*_,*n*_*2*_*}))/(n*_*1*_*+n*_*2*_*)*, and for groupings of three groups by the formula *(max({n*_*1*_,*n*_*2*_,*n*_*3*_*}))/(n*_*1*_*+n*_*2*_*+n*_*3*_*)*

To address our main research question RQ2, we evaluate how well the convolutional neural network model can learn a labeling that matches the grouping. This evaluation is based on computing training and validation metrics of the convolutional neural network model and comparison of the validation accuracy with the probability of pure chance. We carry out machine learning experiments with a basic implementation of a convolutional neural network algorithm that we run in a TensorFlow programming environment [39].

### 2. Guidance about giving the “need for help” ratings for expression statements

Before the online questionnaire started to collect actual answers, the person was provided with the following guidance texts about how he/she should perform the interpretation tasks: *“We ask you to evaluate different expressions, for example the expression ‘I am happy’. Interpret how much each expression tells about the need for help. Give your interpretation about the expression on a numeric scale 0-10. 0 indicates the smallest possible need for help and 10 indicates the greatest possible need for help.“* Then a small training phase allowed the person to get accustomed to give the “need for help” ratings by rating three expression statements: “I have a good health condition.”, “I have a bad health condition.” and “I have an ordinary health condition.” The answers that the person gave during the training phase were excluded from the data set that we use in the analysis reported in this our current research article.

After the training phase, the person was provided with the following guidance texts to still further clarify how he/she should perform the interpretation tasks: *“Do not interpret how much the expression tells about just your own situation. Instead, interpret what kind of impression this expression induces in you. Thus give your interpretation about the expression’s meaning in respect to the mentioned property.”* After showing those guidance texts, the person was allowed to start giving actual questionnaire answers, i.e. to perform the actual interpretation tasks.

### 3. Formulation of the questionnaire items

**Fig. 2:**
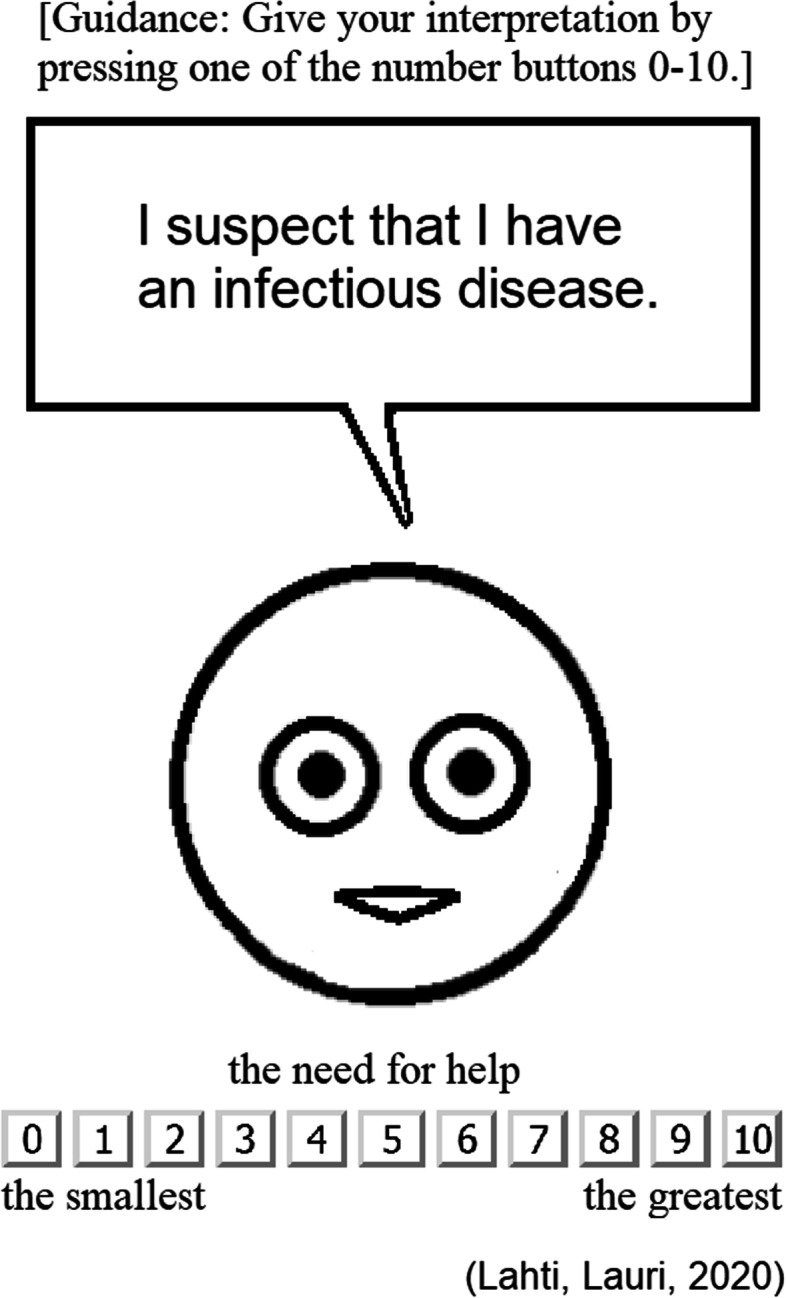
Gathering the “need for help” rating for an expression statement on an 11-point Likert scale with an online questionnaire

In the interpretation tasks, our online questionnaire asked the person to give a rating of the “need for help” for twenty expression statements (ES) that we had extracted with the method we developed and reported in our previous research [40] from the official national guidelines of Finnish Institute for Health and Welfare (THL) [41] and international guidelines of World Health Organization (WHO) [42] concerning the coronavirus COVID-19 epidemic. These twenty expression statements ES1-ES20 included among others descriptions of possible symptoms of the coronavirus, how to deal mild cases of the coronavirus with just self-care, when one should seek admission for professional care and what kinds of practicalities are suggested as a prevention (see Table 2). The expression statements were shown, one at a time, in a speech bubble above a simple briefly animating face figure that remained the same for all the expression statements (see Fig. 2 and further details in Data analysis supplement (Additional file 1)).

Furthermore, the person was asked to answer to nine background questions (BQ, see Table 3). These gathered four answers concerning his/her evaluation about own health, quality of life, and satisfaction about health and ability, responded on a 9-point Likert scale (BQ1 and BQ5-BQ7, adapted from [32, 35–37]). In addition, binary no/yes answers were gathered to questions asking if a health problem reduces the person’s ability (BQ2) and if he/she has a continuous or repeated need for a doctor’s care (BQ4) (adapted from [32]). The person was also asked to tell his/her sex (BQ8) and age (BQ9) and to indicate if a doctor had identified one or more diseases in him/her and to describe them (BQ3) (in a form adapted from [32]).

We have gathered questionnaire answers in Finnish language but we now report our results in English (see original Finnish texts in Data analysis supplement (Additional file 1)). Due to inherent linguistic and cultural differences we assume that the semantic meanings in the translated English versions of expression statements cannot fully match with the original Finnish meanings. On the other hand, we have aimed to follow carefully also those adapted Finnish translations that have been used already earlier in Finnish national health surveys [32, 37].

### 4. Formulation of machine learning experiments

To address our main research question RQ2, we carried out machine learning experiments with a basic implementation of a convolutional neural network algorithm that we run in a TensorFlow programming environment (adapted from TensorFlow image classification tutorial [39]). Our approach consisted of creating an image classifier using a *keras.Sequential* model with *layers.Conv2D* layers and then providing input data to the model in the form of images. We used a model consisting of three convolution blocks with a max pool layer in each of them and having on the top a fully connected layer that is activated by a *relu* activation function. We compiled our model with the *optimizers.Adam* optimizer and the *losses.SparseCategoricalCrossentropy* loss function. Table 5 describes layers of the convolutional neural network model used in the machine learning experiments.

**Table 5:** Layers of the convolutional neural network model used in the machine learning experiments

Model: “sequential” *Parameters*: total 73,112; trainable: 73,112; non-trainable: 0		
*Layer (type)*	*Output shape*	*Number of parameters*
rescaling_1 (Rescaling)	(None, 20, 25, 3)	0
conv2d (Conv2D)	(None, 20, 25, 16)	448
max_pooling2d (MaxPooling2D)	(None, 10, 12, 16)	0
conv2d_1 (Conv2D)	(None, 10, 12, 32)	4640
max_pooling2d_1 (MaxPooling2D)	(None, 5, 6, 32)	0
conv2d_2 (Conv2D)	(None, 5, 6, 64)	18,496
max_pooling2d_2 (MaxPooling2D)	(None, 2, 3, 64)	0
flatten (Flatten)	(None, 384)	0
dense (Dense)	(None, 128)	49,280
dense_1 (Dense)	(None, 2)	258

Although our decision to use an image classifier requires now an additional transformation step for our initially character-encoded questionnaire data and thus can potentially introduce imprecision for the results, we motivate its use here as a general baseline architecture that can be fed with various alternative input data formats for comparison purposes. Thus by using this currently popular and openly available model we aim to facilitate building comparability of machine learning results across various biomedical data classification tasks containing diverse data formulations, and also enabling a possibility to involve humanly intuitive evaluation about the emerging data patterns from the intermediary raster image representations of labeled data sets.

Since the convolutional neural network model required labeled input data in the form of images, we transformed with a self-made R language script our originally character-encoded questionnaire data into a set of grayscale raster images before feeding it to the model.

First the original rating answer values in the range 0-10 were transformed linearly into the range 0.0-1.0. Each entity of twenty rating answers (in the range 0.0-1.0) of expression statements ES1-ES20 given by a certain person were transformed into an individual raster image so that each single rating answer value was represented by a region of 25 pixels (width 5 pixels and height 5 pixels) having a brightness value in the range 0-255 directly proportional to the greatness of the transformed answer value in the range 0.0-1.0. All the twenty separate 25-pixel-sized regions were then joined as a 5 × 4 matrix to form a combined grayscale raster image (width 25 pixels and height 20 pixels).

We performed machine learning experiments with labeled images so that their labeling matched the groupings that we have just previously analyzed with Wilcoxon rank-sum test (i.e., Mann–Whitney U test) between two groups and Kruskal-Wallis test between three groups to identify statistically significant rating differences (see Table 4). We allocated for the training and validation of the machine learning model 80% and 20% of the data, respectively.

Our chosen basic implementation of a convolutional neural network algorithm [39] enables to evaluate the general applicability of machine learning approach in this knowledge context. We have chosen this specific implementation of a convolutional neural network for our experiments since this model is openly and easily available for testing purposes in a currently popular programming environment and the model’s internal computational logic is clearly documented. We use this model as a baseline architecture to gain measures of the performance of machine learning that enable comparison between our parallel data subsets as well as offer our current results to be compared later with future experiments in a well-documented way. We train the machine learning model with the same groups that we use to identify statistically significant rating differences, and this offers insight about how the dependencies between ratings and background information can influence the results of machine learning. Based on the gained findings we then make some conclusions motivated by the previous research and discuss about implications for developing the methodology for interpretation of the patient’s expressions to support his/her personalized care.

It needs to be emphasized that we evaluate the general applicability of machine learning approach for interpretation of the patient’s expressions now in such a way that our current highest developmental priority is *not* to reach a model that manages to learn to detect given groupings very well. Instead, our current highest developmental priority is to propose and experimentally motivate a new methodology that we have developed for evaluating how machine learning results depend on various properties of the data which can be inspected and identified with traditional statistical methods. Thus due to the overall complexity of modeling semantics of a natural language and the limited size of the current data set our gained results are *not* meant to introduce a model that can actually learn the groupings very well. Instead, we aim to introduce now a new methodology that can be used for analyzing how the machine learning models are influenced by the properties of the data so that these notions can be exploited to develop better human-understandable machine learning and furthermore to help to address the traditional challenges of interpreting reliably and intuitively machine learning results [11].

## Results

### 1. Addressing the main research question RQ1

#### 1.1 Identifying statistically significant rating differences for expression statements in respect to background questions

In accordance with Table 1, our proposed new methodology in respect to the step 2 consists of identifying statistically significant and non-significant differences for expression statements in respect to groupings based on the answer values of background questions (for example groupings relying on the person’s answer about his/her estimated health condition).

### 1.1.1 Participants and stages

 We carried out a quantitative cross-sectional study with only one stage (n = 673).

### 1.1.2 Descriptive data

 We gained a diverse distribution of answer values for the background questions (n = 673). Table 6 shows the frequencies of persons giving the answer values 1-9 for the background questions BQ1 and BQ5-BQ7. For example the mean answer value for an estimated health condition (BQ1) was 6.53 (SD=1.97). Table 7 describes the distribution of answer values for the background questions BQ2-BQ4 and BQ8-BQ9. For example 67% of the respondents indicated that a health problem reduces ability (BQ2) whereas 33% did not (M = 1.67; SD=0.47; No coded as 1, Yes coded as 2).

**Table 6:** Frequencies of persons giving the answer values 1-9 for the background questions BQ1 and BQ5-BQ7 (n = 673). M = mean, Mdn=median, SD=standard deviation

*Background question (BQ)*	*Answer value*											
	*1*	*2*	*3*	*4*	*5*	*6*	*7*	*8*	*9*	*M*	*Mdn*	*SD*
BQ1: an estimated health condition	11 (2%)	3 (~0%)	40 (6%)	66 (10%)	98 (15%)	45 (7%)	162 (24%)	129 (19%)	119 (18%)	6.53	7	1.97
BQ5: the quality of life	7 (1%)	6 (1%)	29 (4%)	47 (7%)	101 (15%)	84 (12%)	187 (28%)	123 (18%)	89 (13%)	6.53	7	1.77
BQ6: the satisfaction about health	17 (3%)	10 (1%)	68 (10%)	61 (9%)	84 (12%)	78 (12%)	151 (22%)	138 (21%)	66 (10%)	6.13	7	2.04
BQ7: the satisfaction about ability	8 (1%)	9 (1%)	44 (7%)	28 (4%)	54 (8%)	58 (9%)	156 (23%)	128 (19%)	188 (28%)	6.98	7	1.98

**Table 7:** The distribution of answer values for the background questions BQ2-BQ4 and BQ8-BQ9. M = mean, Mdn=median, SD=standard deviation

*Background question (BQ)*	*Answer value*
BQ2: a health problem reduces ability	No (coded as 1): 219 (33%); Yes (coded as 2): 454 (67%) (M = 1.67; Mdn=2; SD=0.47)
BQ3: one or more diseases identified by a doctor	Disease category (the number of unique persons who selected the category): Lung diseases: 126; Heart and circulatory diseases: 177; Joint and back diseases: 301; Injuries:103; Mental health problems: 188; Vision and hearing deficits: 191; Other diseases: 345
BQ4: a continuous or repeated need for a doctor’s care	No (coded as 1): 364 (54%); Yes (coded as 2): 309 (46%) (M = 1.46; Mdn=1; SD=0.50)
BQ8: the sex	Man (coded as 1): 123 (18%); Woman (coded as 2): 550 (82%) (M = 1.82; Mdn=2; SD=0.39)
BQ9: the age	Belonging to an age range category (the lower bound is included in the range but not the upper bound): 16-20 years: 143 (21%); 20-30 years: 21 (3%); 30-40 years: 61 (9%); 40-50 years: 96 (14%); 50-60 years: 135 (20%); 60-70 years: 141 (21%); 70-80 years: 64 (10%); 80-90 years: 12 (2%); 90 years or more: 0 (0%) (M = 46.93; Mdn=51; SD=19.57)

#### 1.1.3 Outcome data, main results and other analyses

**Fig. 3:**
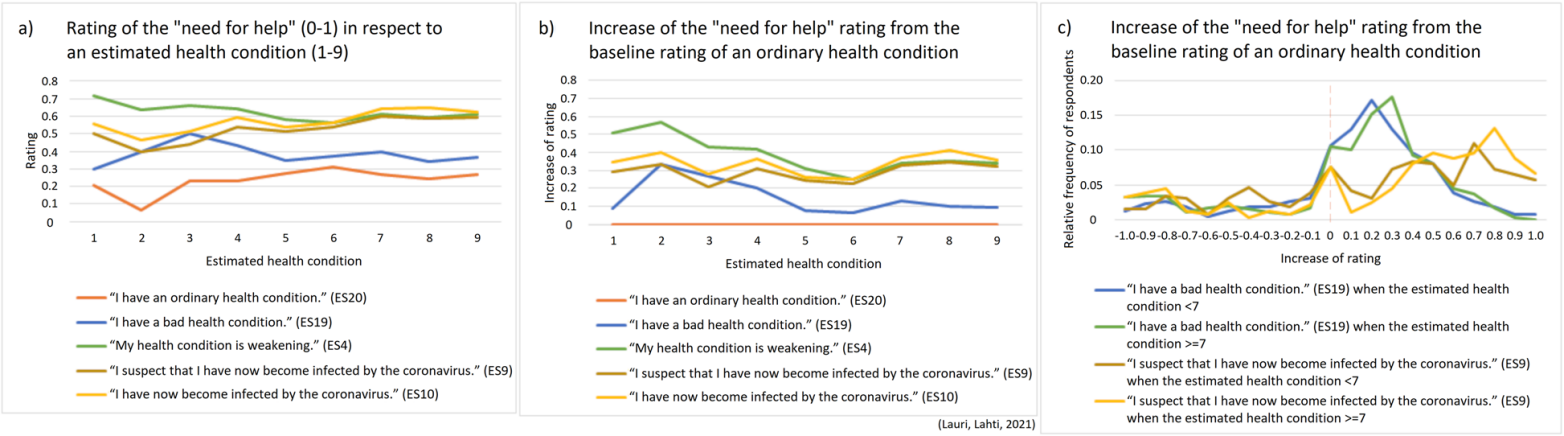
**a** The “need for help” rating mean values (transformed into the range 0.0-1.0) for expression statements ES4, ES9-ES10 and ES19-ES20 in respect to the person’s answer value to the background question BQ1 (an estimated health condition, 1-9), n = 673. **b**-**c** Increase of the “need for help” rating mean values from the baseline rating mean value that the person gives for the expression statement ES20 (“I have an ordinary health condition.”), n = 673

**Fig. 4:**
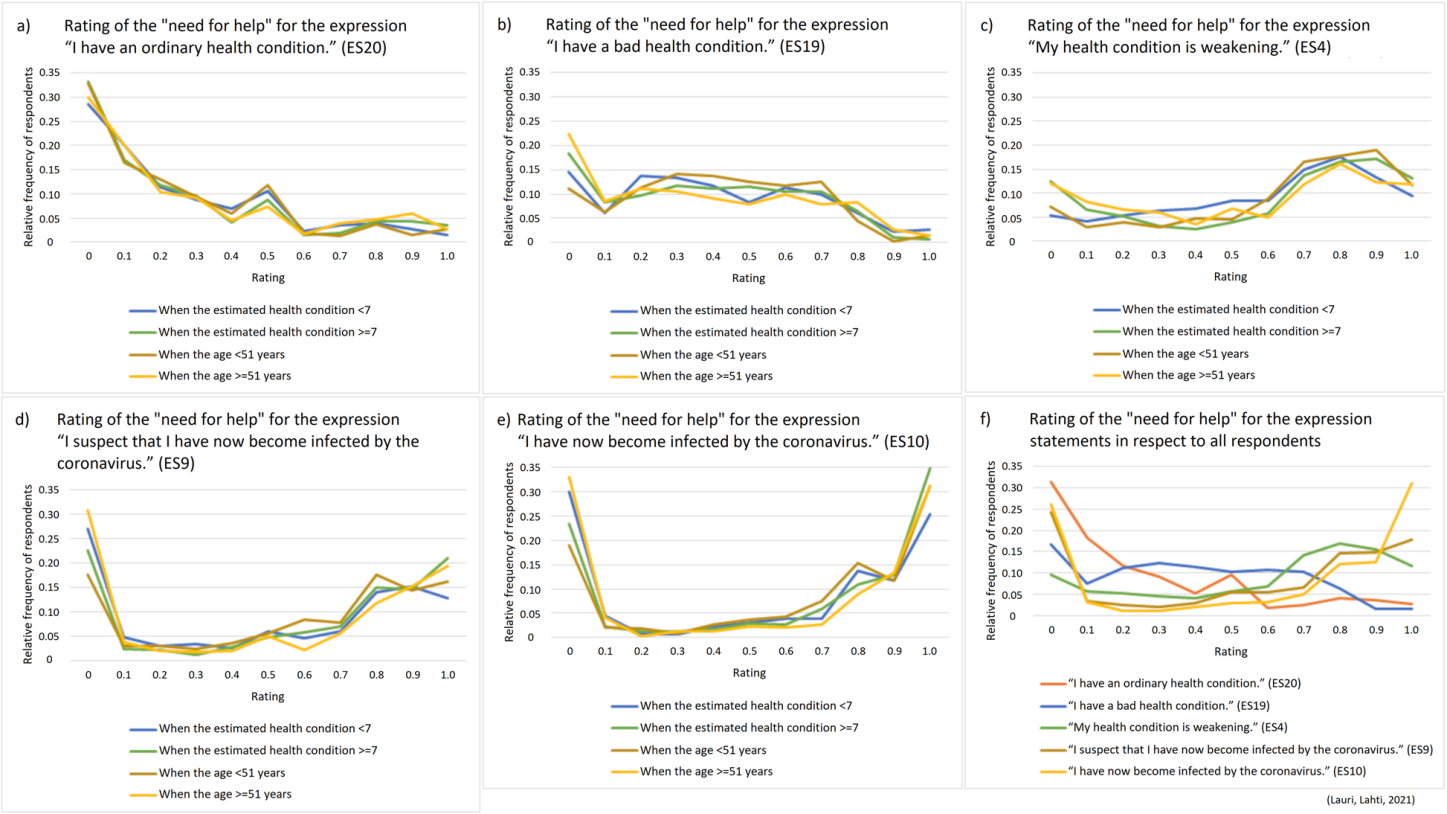
**a**-**e** The relative frequency of respondents for each alternative “need for help” rating value (transformed into the range 0.0-1.0) concerning expression statements ES4, ES9-ES10 and ES19-ES20 in respect to the person’s answer value to the background questions BQ1 (an estimated health condition) and BQ9 (the age), n = 673. **f** Rating value distributions for the expression statements ES4, ES9-ES10 and ES19-ES20 in respect to all respondents together, n = 673

 Figures 3 and 4 show for five expression statements ES4, ES9-ES10 and ES19-ES20 how the “need for help” ratings depend on the person’s answer value to the background question BQ1 that is the person’s estimation about his/her health condition. Figure 3a shows rating mean values for the nine separate groups of respondents corresponding to each possible answer alternative about the estimated health condition (in the range 1-9). Figure 3b and c show the increase of the “need for help” rating mean values from the baseline rating mean value of ES20. On the other hand, Fig. 4 illustrates in a more detail the distribution of the relative frequency of respondents for each alternative rating value in the range 0.0-1.0, in respect to the background questions BQ1 and BQ9.

As shown in Table 8, when computing Kendall rank-correlation measures we found significant correlation (>=0.70 with the level p < 0.001; see [38]) for seven pairs of expression statements and a pair of background questions, all these were statistically significant with the level p < 0.001, and the highest cosine similarity values included the same seven pairs of expression statements and the pair of background questions.

**Table 8:** Pairs of expression statements (ES) and background questions (BQ) having significant correlation (>=0.70 with the level p < 0.001; see [38]) based on a Kendall rank-correlation measure, all these were statistically significant with the level p < 0.001, and the highest cosine similarity values including the same pairs of expression statements and background questions

*A pair of expression statements (ES) and background questions (BQ)*	*Kendall rank-correlation measure*	*Cosine similarity measure*
ES16&ES17	0.91	0.97
ES14&ES15	0.86	0.95
ES9&ES10	0.79	0.92
ES16&ES18	0.78	0.90
ES17&ES18	0.77	0.89
ES7&ES8	0.75	0.87
ES1&ES2	0.73	0.80
BQ1&BQ6	0.71	0.82

A significant correlation (>=0.70 with the level p < 0.001; see [38]) linked expression statements in five thematic subentities which are: an infectious disease (suspecting to have an infectious disease, having it, or having it with a doctor’s verification; ES16-ES18), a lack of coping independently (a lack of coping independently in everyday life or at home; ES14-ES15), the coronavirus (suspecting to have the coronavirus infection or having it; ES9-ES10), a fever (having a fever or a sudden rise of fever; ES7-ES8), and a flu/cough (having a flu or a cough; ES1-ES2). Furthermore, a significant correlation (>=0.70 with the level p < 0.001; see [38]) linked background questions in a thematic subentity about health (an estimated health condition or the satisfaction about health; BQ1&BQ6).

The highest cosine similarity measure values emerging among the same value pairs seemed to support the clusters just identified by the correlation. This same highest cosine similarity measure value range (>=0.80) was reached also by the following pairs: having a sudden rise of fever and suspecting to have the coronavirus infection (ES8&ES9, 0.87), having a sudden rise of fever and having the coronavirus infection (ES8&ES10, 0.86), having a shortness of breath and a weakening health condition (ES3&ES4, 0.83), having the coronavirus infection and having an infectious disease with a doctor’s verification ES10&ES17 (0.82), suspecting to have the coronavirus infection and having an infectious disease with a doctor’s verification (ES9&ES17, 0.81), having the coronavirus infection and having an infectious disease (ES10&ES16, 0.80) and the quality of life and the satisfaction about health (BQ5&BQ6, 0.80).

Wilcoxon rank-sum test (i.e., Mann–Whitney U test) between two groups and Kruskal-Wallis test between three groups indicated statistically significant rating differences for expression statements ES1-ES20 in respect to groupings based on the answer values of each background question (BQ), as shown in Table 4. Table 4 shows also the differences of mean ratings for the groupings. For example, for ES4 (having a weakening health condition) the younger respondents gave a mean rating value 0.66 that was 0.10 greater than the mean rating value 0.56 given by the older respondents (BQ9, for two groups).

Supplementing tests of one-way analysis of variance (ANOVA) between two groups and between three groups indicated statistically significant rating differences largely for the same expression statements as Wilcoxon rank-sum test and Kruskal-Wallis test. However, this statistical significance did not reappear with ANOVA tests between groups for ES5 in respect to BQ1 for three groups, ES14 in respect to BQ2 for two groups, ES19 in respect to BQ9 for three groups, and ES20 in respect to BQ5 for three groups. ANOVA tests between groups indicated also some additional statistically significant rating differences, such as for ES9-ES10 and E17 in respect to BQ2 for two groups, ES9-ES10 in respect to BQ9 for two groups, and ES4 in respect to BQ7 for two groups.

A complete listing of means, medians and standard deviations of the “need for help” ratings for the groupings is provided in Data analysis supplement (Additional file 1) which includes also a comprehensive listing of Kendall rank-correlation and cosine similarity measures, and tests of Wilcoxon rank-sum, Kruskal-Wallis and one-way analysis of variance (ANOVA) between groups.

**Fig. 5:**
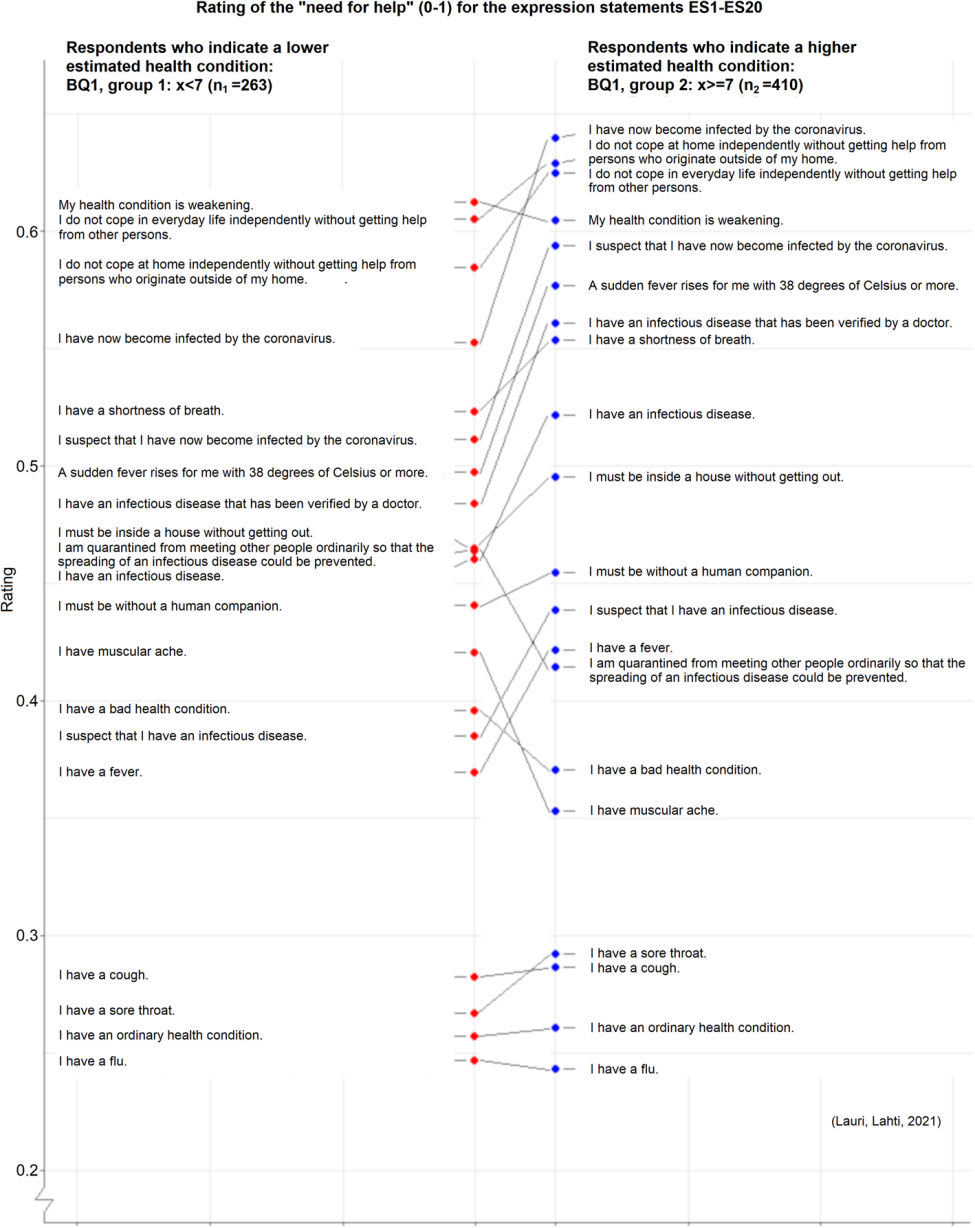
The “need for help” rating mean values of expression statements ES1-ES20 (transformed into the range 0.0-1.0) in respect to two groups based on the answer values of the background question BQ1 (an estimated health condition, 1-9). The “group 1” contains those respondents who gave an answer value that was lower than 7 (n_1_=263), and the “group 2” contains all the other respondents (n_2_=410)

Figure 5 illustrates for all the twenty expression statements ES1-ES20 how the “need for help” rating mean values differ between the respondents who indicate a lower estimated health condition and the respondents who indicate a higher estimated health condition (BQ1, for two groups). Besides comparing just single expression statements between groups, we can now also identify the emergence of two different ranking orders for all the twenty expression statements ES1-ES20 in respect to the grouping based on the answer values of the background question BQ1.

### 2. Addressing the main research question RQ2

#### 2.1 Training and validation of a machine learning model to learn groupings concerning the ratings

In accordance with Table 1, our proposed new methodology in respect to the step 3 consists of training and validation of a machine learning model (with a supervised learning approach) to learn the groupings concerning the “need for help” ratings. This step uses the same groupings of respondents that have been used in the step 2.

Table 4 shows our results about training and validation of the convolutional neural network model to learn a labeling that matches the grouping based on the answer values of each background question, among questions BQ1-BQ2 and BQ4-BQ9 (n = 673). For each grouping we report training and validation metrics gained at such an epoch step when we reached the lowest value for the validation loss (ensured by further 50 evaluation steps with a patience procedure), averaged from 100 separate training and validation sequences.

**Fig. 6:**
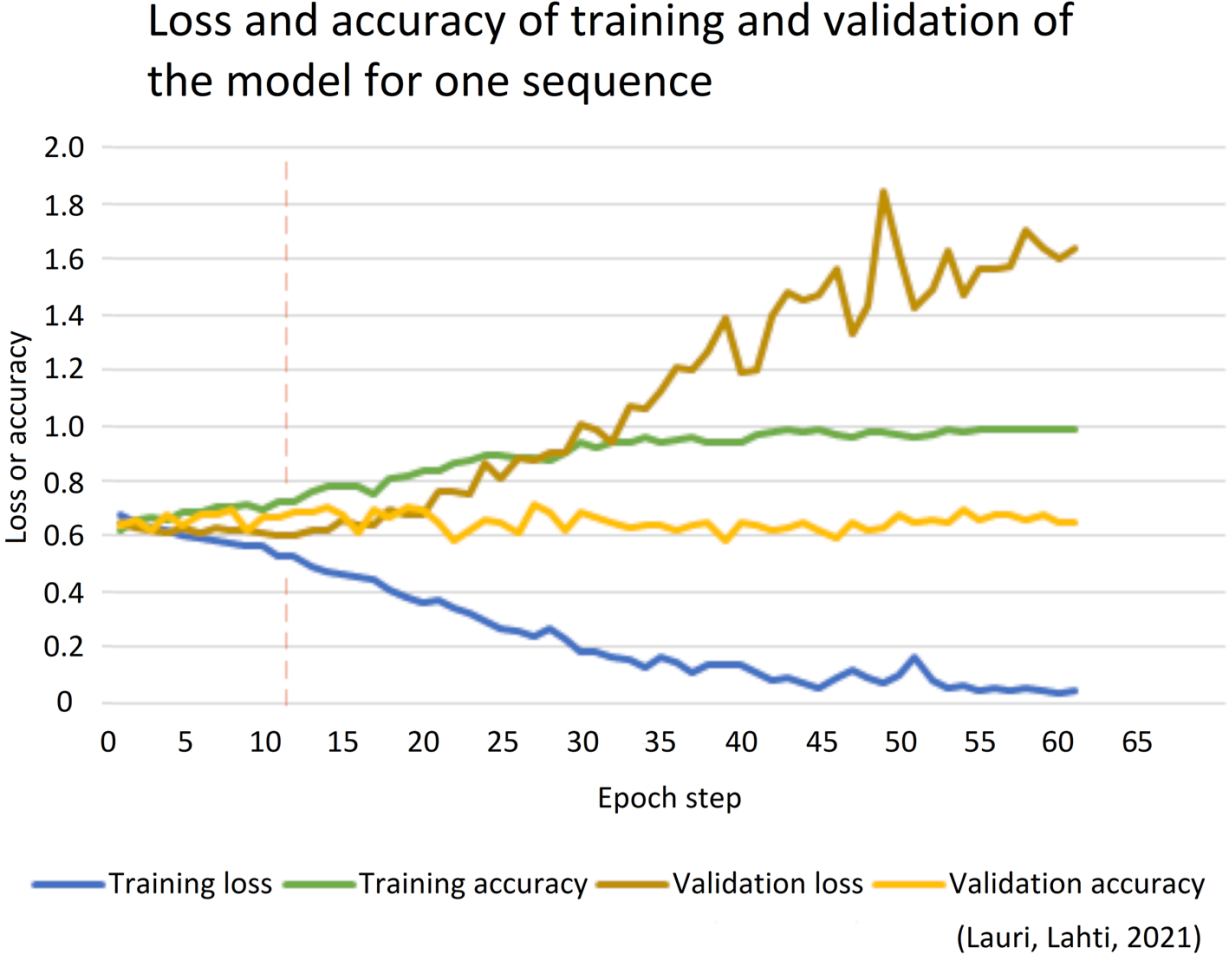
Loss and accuracy for training and validation of the convolutional neural network model for one sequence to learn a labeling that matches the grouping of two groups based on the answer values of the background question BQ1 (an estimated health condition) (n = 673)

Figure 6 illustrates the loss and accuracy for training and validation of the convolutional neural network model for one sequence to learn a labeling that matches the grouping of two groups based on the answer values of the background question BQ1 (an estimated health condition) (n = 673). In this illustrated single sequence the lowest value for the validation loss was reached at the epoch step 11 and at that step the following metrics were gained: training loss 0.53, training accuracy 0.73, validation loss 0.60 and validation accuracy 0.67.

#### 2.2 Comparing the validation accuracies of the machine learning model with the probabilities of pure chance

In accordance with Table 1, our proposed new methodology in respect to the step 4 consists of comparing the validation accuracies of the machine learning model with the probabilities of pure chance of classifying the rating profiles correctly corresponding to groupings relying on the answer values of each background question (averaged from at least 100 separate training and validation sequences). The probability of pure chance of classifying the rating profiles correctly is computed by dividing the size of the greatest group of the grouping (n_1_, n_2_ or n_3_) by the number of all respondents (n = 673). Please see in Table 4 the two most right-sided columns. Then it is possible to compute the difference of the mean validation accuracy and the probability of pure chance of classifying the rating profiles correctly corresponding to each grouping. Since the limited rating value range and the non-continuous stepping of rating values did not allow us to divide the respondents into equally-sized groups, we used for the probability of pure chance a formula which has the size of the greatest group of the grouping as the numerator. To be on the safe side, we used this conservative formulation but we suggest that the probability of pure chance could be computed also with an alternative formulation that can possibly enable reaching a greater difference of the mean validation accuracy and the probability of pure chance than when using the conservative formulation.

As Table 4 shows, the difference of the mean validation accuracy and the probability of pure chance of classifying the rating profiles correctly has the highest values for the groupings of two groups which are “BQ9, two groups” (0.17), “BQ1, two groups” (0.08) and “BQ6, two groups” (0.07). Furthermore, the difference has the highest values for the groupings of three groups which are “BQ9, three groups” (0.16), “BQ6, three groups” (0.03) and “BQ1, three groups” (0.03).

#### 2.3 Contrasting the validation accuracies of the machine learning model with the statistically significant rating differences in respect to groupings

.

To describe our proposed new methodology in accordance with Table 1, the just mentioned step 4 is closely linked with the step 5. The step 5 consists of contrasting the validation accuracies of the machine learning model with the occurrence of statistically significant and non-significant rating differences for expression statements in respect to groupings based on the answer values of background questions (averaged from at least 100 separate training and validation sequences). We propose that this contrasting can be done intuitively by evaluating various properties of the rating differences concerning the expression statements for each grouping. These properties can include the frequencies, the strengths (levels) of statistical significance, rankings and distributions of the rating differences. We now illustrate this evaluation approach for the grouping “BQ1, two groups” as shown in Table 4.

For the grouping “BQ1, two groups” statistically significant rating differences emerge for eight expression statements which are ES6-ES10 and ES16-ES18. Among them ES6 has a statistical significance with the highest level that is p = 0.001, ES8-ES10 have a statistical significance with the second highest level p < 0.01 and the remaining ES7 and ES16-ES18 have a statistical significance with the third highest level p < 0.05. Already these notions enable to identify rankings and distributions of the rating differences for expression statements in respect to the grouping “BQ1, two groups” based on the decreasing order of statistical significance (e.g., ES6 having the highest level) and the pattern of semantic topics of the expression statements that belong to the subset of eight expression statements that now reached statistical significance among all the 20 expression statements. Further rankings and distributions can be identified based on the values of rating differences for expression statements, for example ES6 having the highest positive rating difference value (0.07) and ES10 having the lowest negative rating difference value (-0.09), and for example the absolute values of each of the eight statistically significant rating differences being in the range of [0.05, 0.09] in a specific decreasing order.

#### 2.4 Drawing conclusions about the applicability of the current machine learning model

In accordance with Table 1, in our proposed new methodology a specific role is reserved for the step 6. The step 6 consists of drawing conclusions about the applicability of the current machine learning model in this knowledge context. Based on the conclusions further fitting can be done for the model and then it is possible to iteratively repeat the steps 2-6. Since the distributional properties of the questionnaire answers can vary extensively in different cases of using the methodology, it is thus challenging to offer now any comprehensive description about the principles how the conclusions should be drawn preferably in a general case and how the fitting of the model and iterative evaluation could be addressed suitably. Therefore relying on the previous research and our new experimental results, we now suggest that a general guideline for carrying out the step 6 is to emphasize parallel and complementing data analysis methods so that initial weaker findings could become gradually more verified with cumulative further analysis that cross-examines the identified dependencies and influences. Anyway, our results reported in Table 4 motivate an illustration of empirical application of the step 6 in the current case of using the methodology with our gathered experimental data that has a limited size.

## Discussion

### 1. Emerging statistically significant dependencies and influences

In accordance with the steps 1-6 of Table 1, motivated by the previous research and based on our gained findings we now discuss about implications for developing the methodology for interpretation of the patient’s expressions to support his/her personalized care. The steps 1-2 of Table 1 are addressed by the main research question RQ1. In respect to our main research question RQ1, we have analyzed how different people rate the “need for help” for expression statements concerning imagined care situations related to the coronavirus COVID-19 epidemic and how this rating depends on the background information about the person.

For different expression statements the “need for help” ratings have varied distributions as illustrated in Fig. 4. It appears that some expression statements, such as ES10 (having the coronavirus infection), get U-shaped rating distributions which can originate from various reasons worth further future investigation. We currently suggest that the extreme sides of U-shaped rating distributions can possibly indicate that certain respondents interpret even relatively calm situations as strongly threatening (perhaps this is due to having a personality trait/state that easily exhibits anxiousness) and that certain other respondents interpret even relatively threatening situations as strongly calm (perhaps this is due to having a personality trait/state that easily exhibits resilience, or alternatively carelessness or hopelessness). It is also possible that some extreme answers indicate that the person has misunderstood the given interpretation task.

We identified statistically significant rating differences for expression statements in respect to groupings based on the answer values of each background question, between two groups and between three groups (with Wilcoxon rank-sum test and Kruskal-Wallis test, respectively), as shown in Table 4. Supplementing tests of one-way analysis of variance (ANOVA) between groups also largely supported these findings, and indicated even some other statistically significant rating differences. To keep our analysis compact, we now discuss about the statistically significant rating differences especially in respect to Wilcoxon rank-sum test and Kruskal-Wallis test but similar notions apply well also in respect to ANOVA tests between groups (see further details in Data analysis supplement (Additional file 1)).

In groupings of two groups, the highest number of statistically significant rating differences (p < 0.05) emerged for the expression statements ES11 (to be quarantined from meeting other people to prevent spreading an infectious disease, 7 groupings) and ES6 (having muscular ache, 6 groupings). The rating for ES11 differed statistically significantly for all the background questions, except BQ1 (an estimated health condition), between two groups (lower answer values vs. higher answer values). The mean rating of ES11 was higher when getting lower answer values to BQ5-BQ7 (“group 1”; the quality of life, the satisfaction about health, the satisfaction about ability) than when getting higher answer values to BQ5-BQ7 (“group 2”). In contrast, the mean rating of ES11 was lower when getting lower answer values to BQ2, BQ4, BQ8 and BQ9 (“group 1”; a health problem reduces ability, a continuous or repeated need for a doctor’s care, the sex, the age) than when getting higher answer values to BQ2, BQ4, BQ8 and BQ9 (“group 2”).

Since ES11 refers to an essential coronavirus-related situation (to be quarantined from meeting other people to prevent spreading an infectious disease), this emerging high differentiation of the “need for help” ratings can be considered as an important new finding that should be addressed when interpreting a person’s need for help during an epidemic (such as the coronavirus COVID-19 epidemic). Further research is needed to better confirm this our new finding but meanwhile we provide some initial illustration about the statistically significant rating differences for ES11 in respect to groupings based on the answer values of background questions. For example, the respondents who indicated a lower quality of life (BQ5, two groups) gave for ES11 a mean rating of 0.47, whereas the respondents who indicated a higher quality of life gave a mean rating of 0.41. On the other hand, the respondents who indicated a lower age (BQ9, two groups) gave for ES11 a mean rating of 0.41, whereas the respondents who indicated a higher age gave a mean rating of 0.46.

Besides groupings of two groups, ES11 (to be quarantined from meeting other people to prevent spreading an infectious disease) and ES6 (having muscular ache) gained the highest number of statistically significant rating differences (p < 0.05) also in respect to groupings of three groups (4 groupings for both ES11 and ES6). Other expression statements having a high number of statistically significant rating differences in groupings of two or three groups include ES8-ES10 (having a sudden rise of fever, suspecting to have the coronavirus infection or having it, 5 or 6 groupings). Since ES8-ES10 refer to an essential coronavirus-related situation, also this emerging high differentiation of the “need for help” ratings can be considered as an important new finding that should be addressed when interpreting a person’s need for help, for example to support personalized screening, diagnosis and care planning. These three expression statements ES8-ES10 gained lower mean ratings from respondent groups who indicated a lower estimated health condition (BQ1), a lower quality of life (BQ5) and being a man (BQ8), and higher mean ratings from the opposite groups, respectively.

Statistically significant rating differences (p < 0.05) in groupings of two groups emerged the most for the background question BQ8 (the sex, 13 expression statements), then followed by BQ9 (the age, 12), BQ1 (an estimated health condition, 8), BQ5 (the quality of life, 6), BQ2 (a health problem reduces ability, 5), BQ7 (the satisfaction about ability, 3), BQ4 (a continuous or repeated need for a doctor’s care, 2), and BQ6 (the satisfaction about health, 2). Relatively similarly, in groupings of three groups, statistically significant rating differences (p < 0.05) emerged the most for the background question BQ9 (13 expression statements), then followed by BQ5 (7), BQ1 (5), BQ6 (2), and BQ7 (2).

Figure 5 illustrates the emergence of two different ranking orders for the “need for help” ratings of expression statements ES1-ES20 in respect to the grouping based on the answer values of the background question BQ1 (an estimated health condition) for two groups. Already these kinds of rankings can assist in addressing the needs of the patient depending on his/her background information. For example based on our results, for ES4 (having a weakening health condition) the younger respondents (BQ9, for two groups) gave a mean rating value 0.66 that was 0.10 greater than the mean rating value 0.56 given by the older respondents. This our finding can indicate that when seeking admission to care a representative of the younger people may interpret the need for help concerning this expression statement differently than a representative of the older people. To prevent misunderstandings and malpractices it is important to be aware of such possible interpretational differences in communication and decision making about care.

The “need for help” ratings can be exploited also in many other ways to create rankings that can support personalizing the care. Each background question is linked to a specific set of expression statements (if any) that show statistically significant rating differences for this background question. Based on the rating differences and their strengths (levels) of statistical significance, a ranking order can be identified for those expression statements that are linked to by the same background question. On the other hand, an expression statement can get different rating differences and strengths (levels) of statistical significance for different background questions (if any). This enables to identify for each expression statement a ranking order of background questions that link to it.

These various ranking orders offer an opportunity to find some distinctive link patterns between the person’s “need for help” ratings for expression statements and his/her answer values to background questions, and vice versa. For example, in groupings of two groups, ES14 (a lack of coping independently in everyday life) and ES15 (a lack of coping independently at home) show statistically significant rating differences for BQ2 (a health problem reduces ability, 0.06 and 0.08, respectively) but not for BQ5 (the quality of life), and on the other hand ES16 (having an infectious disease) and ES17 (having an infectious disease with a doctor’s verification) show statistically significant rating differences for BQ5 (-0.06 and -0.07, respectively) but not for BQ2. This emerging differentiation may enable a conclusion that the “need for help” ratings about coping independently (ES14-ES15) are more closely linked to having a health problem that reduces ability (BQ2) than to the quality of life (BQ5). Similarly, it may be concluded that the “need for help” ratings about an infectious disease (ES16-ES17) are more closely linked to the quality of life (BQ5) than to having a health problem that reduces ability (BQ2).

After just discussing about the steps 1-2 of Table 1, we now continue to discuss about the steps 3-6. The steps 3-6 of Table 1 are addressed by the main research question RQ2. In respect to our main research question RQ2, we performed machine learning experiments with the answer value sets transformed to labeled raster images so that their labeling matched the groupings that we have just previously analyzed with Wilcoxon rank-sum test and Kruskal-Wallis test (as shown in Table 4). This was motivated by the assumption that machine learning enables more flexibility for modeling than for example logistic regression models of traditional statistics [11]. We trained and validated a convolutional neural network model to learn a labeling that matches the grouping. In groupings of two groups, the highest mean values of validation accuracy emerged for the background question BQ8 (the sex, 0.79), then followed by BQ7 (the satisfaction about ability, 0.72), BQ1 (an estimated health condition, 0.69), BQ9 (the age, 0.68), BQ2 (a health problem reduces ability, 0.66), BQ5 (the quality of life, 0.60), BQ6 (the satisfaction about health, 0.60) and BQ4 (a continuous or repeated need for a doctor’s care, 0.57). In groupings of three groups, the highest mean values of validation accuracy emerged for the background question BQ9 (the age, 0.50), then followed by BQ7 (the satisfaction about ability, 0.47), BQ5 (the quality of life, 0.42), BQ1 (an estimated health condition, 0.40) and BQ6 (the satisfaction about health, 0.39).

### 2. Limitations

As motivated in the chapters “Methods” and “Results”, due to the overall complexity of modeling semantics of a natural language and the limited size of the current data set our gained results are *not* meant to introduce a model that can actually learn the groupings very well. Instead, we aim now to propose and experimentally motivate a new methodology that can be used for analyzing how the machine learning models are influenced by the properties of the data so that these notions can be exploited in the future research to develop better machine learning models. We have chosen the specific openly available implementation of a convolutional neural network (adapted from TensorFlow image classification tutorial [39]) as a baseline architecture to gain measures of the performance of machine learning that enable comparison between our parallel data subsets as well as offer our current results to be compared later with future experiments in a well-documented way.

Since our essential goal is to ensure generating and evaluating comparable measures concerning the machine learning experiments, we do not want to rely just on the value of validation accuracy but instead we preferably want to observe especially the difference of the mean validation accuracy and the probability of pure chance of classifying the rating profiles correctly corresponding to groupings relying on the answer values of each background question (as shown in Table 4). As described in the chapter “Results”, the difference of the mean validation accuracy and the probability of pure chance of classifying the rating profiles correctly has varied values for different groupings and has the highest values for the groupings of two or three groups in respect to the background questions BQ9 (the age), BQ1 (an estimated health condition) and BQ6 (the satisfaction about health) so that the difference values remain clearly above the value zero. Thus at least for the groups of these background questions BQ9, BQ1 and BQ6 the mean values of validation accuracy are clearly above the probabilities of pure chance. This in turn allows us to make a conclusion that in respect to these groupings the machine learning results may have been well-influenced by the properties of the data and possibly especially by such properties that are related to the statistically significant rating differences that we have identified with traditional statistical methods. Due to the limited size of our current data set, it is possible that various dependencies remain now unnoticed. Thus it may be possible that even those groupings that do not now reach such mean values of validation accuracy that are above the probabilities of pure chance can still in future experiments reach them when the size of the data set is increased sufficiently.

Based on our just mentioned notions, we therefore suggest that although the mean values of validation accuracy remained relatively low and only partially above the values of pure chance for the groupings, our machine learning experiments however managed to show the applicability of a baseline convolutional neural network model to support detecting the need for help in the patient’s expressions in respect to groupings relying on the answer values of each background question. Thus especially at least for the groupings relying on the background questions BQ9 (the age), BQ1 (an estimated health condition) and BQ6 (the satisfaction about health) it appears that the machine learning results may be well-influenced by the statistically significant rating differences that we identified for certain specific expression statements, as shown in Table 4. These influences may be especially strong (reaching partially even the statistically significant rating differences of the level p < 0.001) in respect to the “need for help” ratings for expression statements ES1-ES5 (having a flu, a cough, a shortness of breath, a weakening health condition or a sore throat) and ES14-ES15 (a lack of coping independently in everyday life or at home) concerning BQ9, ES6 (having muscular ache) concerning BQ1, and ES11 (to be quarantined from meeting other people to prevent spreading an infectious disease) concerning BQ6. We refer to these four thematic subentities of expression statements as expression sets of possible influence.

Therefore with our current data set in accordance with the step 6 of Table 1, some possible conclusions for further fitting of the model and iterative evaluation of the current baseline machine learning model can include for example adjusting the model’s internal computational logic so that it can better address those certain specific expression statements that have been identified to influence the model’s performance. The model’s adjustments should preferably take into account the particular statistical and semantic properties of these expression statements. These adjustments can consist of among others modification of the model’s layers, filters, pooling, optimizers, activation functions and loss functions. In addition, the adjustments can extend to cover comparing alternative machine learning architectures and their variants and hybrids as well as preprocessing options such as input data formulation and regularization and supplementing statistical or rule-based techniques.

When iteratively evaluating and fitting the machine learning model it is important to seek such a balance that avoids both overfitting and underfitting. For example convolutional neural network models with full connectivity can be prone to overfitting. With our current convolutional neural network model we have aimed to prevent both overfitting and underfitting by stopping the training and validation process at such an epoch step when the model has reached the lowest value for the validation loss and by applying a patience procedure that inspects still some further steps to prevent premature stopping at a local minimum, and also by averaging results from a large amount of separate training and validation sequences. In further experiments we suggest to avoid overfitting also by considering augmenting the original training data set with its random transformations, by dropping out a certain proportion of output units from layers of the model during the training, and by regularization of the input data formulation.

Furthermore, with our current data set the fitting of the machine learning model may benefit from emphasizing especially those expression statements which reached the highest statistically significant rating differences in the four thematic subentities that we identified among them, as discussed above in the chapter “Limitations” (expression sets of possible influence). Thus it may be beneficial to aim at fitting the model to learn the groupings in respect to background questions so that for each grouping the adjustments can address especially those thematic subentities of expression statements that have the highest statistically significant rating differences for this grouping. Thus in the fitting of the baseline model it can be possible to emphasize the following thematic subentities: having respiratory symptoms or a weakening health condition (ES1-ES5) concerning groupings in respect to the age (BQ9); a lack of coping independently (ES14-ES15) concerning groupings in respect to the age (BQ9); having muscular ache (ES6) concerning groupings in respect to an estimated health condition (BQ1); and being quarantined due to an infectious disease (ES11) concerning groupings in respect to the satisfaction about health (BQ6).

### 3. Interpretation of the results

Our just mentioned notions about the applicability of our proposed new methodology are motivated by the previous research that has shown the applicability of an artificial neural network model in identifying the affectivity of online messages about the coronavirus [31] by reaching a testing accuracy of 81.15% in classification that relied on a training set of 338,666 messages and a testing set of 112,888 messages about the coronavirus extracted from the online messaging service Reddit between 20 January and 19 March 2020. Besides having a bigger data set than ours, Jelodar et al. [31] used additional methods of Latent Dirichlet Allocation (LDA) and a pre-existing emotion vocabulary and rules (SentiStrength algorithm) to supplement a Long Short-Term Memory (LSTM) recurrent neural network (RNN) algorithm. In contrast, our results rely purposefully on using just a basic implementation of a convolutional neural network algorithm (TensorFlow image classification tutorial [39]) that we feed with our gathered questionnaire answers (n = 673).

Since we used a relatively small data set of answers and the distributions of some answer values were positioned in a relatively narrow or skewed subrange of the scale range, this may have limited the classification ability of our machine learning model. These partially narrow and skewed distributions have also caused that the probability of pure chance of classifying the rating profiles correctly has varied values for different groupings since that probability is defined based on the size of the greatest group of the grouping (n_1_, n_2_ or n_3_) that reaches varying values for different groupings. This variability in turn has given the motivation that to enable comparability of groupings we want to observe especially the difference of the mean validation accuracy and the probability of pure chance of classifying the rating profiles correctly corresponding to groupings (as shown in Table 4).

Despite the challenges outlined above we have managed to identify some emerging link patterns between our results of machine learning and traditional statistical analysis. For two groups and three groups, the highest mean values of validation accuracy emerged for the background questions BQ8 (the sex) and BQ9 (the age), respectively, which also reached the highest number of statistically significant rating differences (p < 0.05) for expression statements in respect to the same groupings with Wilcoxon rank-sum test and Kruskal-Wallis test (see Table 4). However, the difference of the mean validation accuracy and the probability of pure chance of classifying the rating profiles correctly corresponding to groupings is now clearly above the zero only for the groupings of BQ9 (the age) and not for the grouping of BQ8 (the sex). Thus we suggest making a conclusion that our machine learning results may be influenced by the statistically significant rating differences that we have identified for the groupings of BQ9 but possibly not by the statistically significant rating differences that we have identified for the grouping of BQ8.

We expect that by accumulating a larger data set of answers, it is possible to reach higher values for the difference of the mean validation accuracy and the probability of pure chance of classifying the rating profiles correctly corresponding to groupings. This in turn can enable achieving a more detailed understanding about how the machine learning results depend on and are influenced by the statistically significant rating differences concerning the groupings.

Accumulating knowledge from even sparse data points of diverse single-time interpretative measurements with machine learning gets fruitful support from the previous research that has found relatively good reliability even for single-item observations with increasing efficiency, avoiding confusion and enabling to accumulate answers from people who are hard to reach [43–46]. We now present a new comparative analysis approach to identify and evaluate with traditional statistical methods the dependencies that can explain the machine learning results. Thus our analysis approach enables to develop better human-understandable machine learning and so helps to address the traditional challenges of interpreting reliably and intuitively machine learning results [11]. Therefore, our analysis approach can offer also support for developing reliable evaluation metrics for healthcare chatbots [16] and their ability for semantic understanding [17].

We decided to gather now ratings in respect to the “need for help” since this semantic dimension emerged strongly in the context of health-related online discussions in our previous analysis [40]. However, the selection of the “need for help” dimension can be motivated also by its intuitive relatedness to the dominance dimension [23, 24] that reflects the degree of ability to cope and to be in the control of one’s own life situations, and also to the approach-avoidance dimension [25] that reflects the desire to reach some relieving assistance or to be reached by this assistance.

Our results indicated statistically significant rating differences depending on the person’s sex and age that can be considered to get support from corresponding previous results [24] in which female and older respondents gave on average smaller rating values of pleasure, arousal and dominance than male and younger respondents, respectively, for a diverse set of words. Furthermore, our results concerning statistically significant rating differences depending on the person’s health and wellbeing get support from the previous findings of Warriner et al. [24] in which the most feared medical conditions were also rated to be among the diseases that represent the lowest rating values of pleasure and dominance and the highest rating values of arousal.

To measure the “need for help” ratings the most reliably, the measurements should be done in real-life situations that involve negative experiences but since that is ethically challenging, we now measured the “need for help” with imagined situations. Anyway, experimental setups containing real-life exposure to pain and threats to pain [47] indicated that helplessness correlated highly with rumination and moderately with magnification. Since this previous result has resemblance with our significant correlation (>=0.70 with the level p < 0.001; see [38]) between ratings of suspecting to have the coronavirus infection or having it (ES9-ES10) and between ratings of suspecting to have an infectious disease, having it, or having it with a doctor’s verification (ES16-ES18), this offers support that our measurements of imagined situations can indeed be relatively reliably paralleled with real-life situations. In addition, Berna et al. [26] have found links between self-identified most significant mental imagery describing the patient’s pain and associated triggers, affects, meanings and avoidance patterns.

### 4. Generalizability

Our aim to generalize imaginary-based measurement results to corresponding real-life situations gets also support from the previous findings that the patterns of neural activation during imagery and actual perception have a strong overlap [48–50]. Neuroimaging experiments have indicated that self-report ratings of vividness of mental imagery can correlate with activation of the same sensory-specific cortices as activated in perception [51–53]. Anyway, there is evidence that imagining a future event increases the person’s perception concerning the probability that the imagined event will occur [54, 55]. It has been also shown that people perceive the likelihood of contracting a disease higher when the description of the disease is easier to imagine than when it is harder to imagine [55], and for imagined symptoms people prioritized selecting a simple separate cause than a more complex combination of causes even if the likelihood value for the combination of all the causes was displayed to be higher than for simple separate causes [56]. These previously found adjusting effects on probabilities and prioritization concerning imagining and reasoning may contribute also to the patterns of dependence and influence that we have now identified between our machine learning results and statistically significant rating differences.

Our results can be considered as a supplement to already existing machine learning approaches that have been applied in classification of medical literature, patient records, clinical narratives and patient phenotypes [12–15, 27–29]. However, a specific novelty in our approach is that besides gathering answers about the person’s current real-life situation, we also gathered rating answers that measured the degree of the “need for help” that the person associated with the given imagined care situations. Thus with our “need for help” rating model [21, 22] we developed a new methodology that extracts the person’s behavioral patterns (such as conceptualizations, attitudes and reasonings) associated with various possible future care situations depicted by expression statements. With machine learning these identified behavioral patterns are then linked to certain background information about the person thus enabling to create predictive models. For example, in the context of clinical decision support systems (CDSS), our results can assist in detecting the patient’s need for help and thus enhance reasoning that addresses distinctive and differentiated needs of the patient to enable personalized screening, diagnosis and care planning. Also in self-care and rehabilitation, our results can assist to implement monitoring and recording of the emerging need for help in the person’s everyday life so that necessary assistance can be alerted.

## Conclusions

With our new methodology (see Table 1) statistically significant differences of self-rated “need for help” can be linked to machine learning results. We found statistically significant correlations and high cosine similarity values between various health-related expression statement pairs concerning the “need for help” ratings and a background question pair. We also identified statistically significant rating differences for several health-related expression statements in respect to groupings based on the answer values of background questions, such as the ratings of suspecting to have the coronavirus infection and having it depending on the estimated health condition, quality of life and sex. Our new methodology enabled us to identify how some of the statistically significant rating differences may be linked to machine learning results thus helping to develop better human-understandable machine learning models.

Resembling the previous research that has developed machine learning methods for extracting health-related knowledge [12–15, 27–29] and evaluated the affectivity of online messages about the coronavirus [31], our results offer insight about the applicability of machine learning to extract useful knowledge from health-related expression statements to support healthcare services, such as to provide personalized screening and care. However, to our best knowledge our research is the first of its kind to develop and use the “need for help” rating model [21, 22] to gather self-rated interpretations about health-related expression statements that are then analyzed to identify statistically significant rating differences in respect to groupings based on the answer values of background questions, and then also to show the applicability of machine learning to learn the groupings concerning the ratings. Furthermore, with our new methodology we propose and experimentally motivate how to enable comparable measurements between parallel data subsets as well as for future experiments in a well-documented way. Our results aim to offer resources for developing decision making for personalized care [34].

Our research contribution gets some additional value also from the successful data acquisition process that involved respondents belonging to Finnish patient and disabled people’s organizations, other health-related organizations and professionals, and educational institutions (n = 673) and thus representing a diversity of health conditions, abilities and attitudes. In addition, our results enable to compare the statistically significant rating differences in groupings in respect to the person’s background information and to further contrast them with the training and validation metrics gained in machine learning experiments based on the same groupings (see Table 4). Furthermore we publish an anonymized version of our current research data (the open access data set “Need for help related to the coronavirus COVID-19 epidemic”) in the supplementing spreadsheet file Additional file 2. We also publish additional details about our research methodology, measurements and analysis results in the supplementing document Data analysis supplement (Additional file 1).

Future research should continue exploring and analyzing how different people interpret and evaluate health-related expression statements and how this possibly depends on the person’s background information. A specific emphasis should be given for developing adaptive modular methods that can be flexibly applied for various purposes of health analytics and also enhance fertile standardized practices that ensure comparability. Furthermore, the emerging new models, methods and algorithms should be well human-understandable for everyone and provided with open access, accompanied with appropriately and sufficiently anonymized data sets. In this spirit, we suggest that also our current findings and results can be used as a part of a greater reasoning entity to develop computational methods to identify, interpret and address the needs of the patient in diverse knowledge processes of healthcare to support personalized care.

## Supplementary Information

**Additional file 1.**

**Additional file 2.**

## Data Availability

The data set supporting the conclusions of this article is included within the article and its supplementing document Data analysis supplement (Additional file 1) and open access data set (Additional file 2). While taking appropriate and sufficient anonymization actions in respect to addressing the General Data Protection Regulation of the European Union in handling the research data, DIHEML research project publishes an anonymized version of the current research data (the open access data set “Need for help related to the coronavirus COVID-19 epidemic”) in the supplementing spreadsheet file Additional file 2, to be used by anyone for non-commercial purposes while citing this current research article (Lahti, Lauri, 2022). The first manuscript version of this research article was completed and self-archived on the open access Arxiv repository (https://arxiv.org/abs/2012.13626) on 24 December 2020 with the following author and naming details: “Lahti, Lauri (2020). Detecting the patient’s need for help with machine learning.”

## Abbreviations

ANOVA: analysis of variance
BQ: background question
CDSS: clinical decision support system
COVID-19: coronavirus disease 2019
DIHEML: the research project “Development of method for interpretation of health expressions based on machine learning to support various care events and persons”
ES: expression statement
LDA: Latent Dirichlet Allocation
LSTM: Long Short-Term Memory
M: mean
Mdn: median
RNN: recurrent neural network
RQ: research question
SD: standard deviation
THL: Finnish Institute for Health and Welfare (Terveyden ja hyvinvoinnin laitos, THL)
WHO: World Health Organization
