# Supplementary material for: Detecting the patient’s need for help with machine learning based on expressions
Source: BMC Med Res Methodol. 2022 Mar 6;22:60. doi: 10.1186/s12874-021-01502-8 (PMC8898191; doi:10.1186/s12874-021-01502-8)

Data analysis supplement to the research article "Lahti, Lauri (2022), Detecting the patient's need for help with machine learning based on expressions" to appear in the journal "BMC Medical Research Methodology" (this is Additional file 1 of that research article)

This data analysis supplement is developed and created by Lauri Lahti, Department of Computer Science, Aalto University School of Science, Finland (email: lauri.lahti@aalto.fi), first published on 1 January 2022.

Additional file 1

Supplementary Table 1. Kendall rank-correlation and cosine similarity measures for each comparable pair of parameter values of the "need for help" ratings of expression statements ES1-ES20 and the answers of the background questions BQ1 and BQ5-BQ7 (n=673).
Kendall rank-correlation measures are shown on the upper-right region of the table and cosine similarity measures are shown on the lower-left region of the table.
Before computing cosine similarity measures the answer values of each parameter were normalized by the formula *(x - min(x))/(max(x)-min(x))* and then these new values were shifted so that the mean value was positioned to the zero by the formula *(x - mean(x))*. The statistical significance levels were defined as p<0,05, p<0,01 and p<0,001, denoted by symbols *, ** and ***, respectively.

	ES1	*ES2*	*ES3*	*ES4*	*ES5*	*ES6*	*ES7*	*ES8*	*ES9*	*ES10*	*ES11*	*ES12*	*ES13*	*ES14*	*ES15*	*ES16*	*ES17*	*ES18*	*ES19*	*ES20*	*BQ1*	*BQ5*	*BQ6*	*BQ7*
ES1		0.73 ***	0.40 ***	0.32 ***	0.55 ***	0.40 ***	0.40 ***	0.33 ***	0.26 ***	0.26 ***	0.16 ***	0.22 ***	0.24 ***	0.23 ***	0.22 ***	0.24 ***	0.23 ***	0.28 ***	0.36 ***	0.16 ***	-0.01	-0.01	-0.03	-0.03
ES2	0.80		0.56 ***	0.43 ***	0.64 ***	0.45 ***	0.51 ***	0.43 ***	0.38 ***	0.36 ***	0.19 ***	0.30 ***	0.30 ***	0.32 ***	0.31 ***	0.31 ***	0.30 ***	0.34 ***	0.40 ***	0.07 *	0.00	0.00	-0.01	-0.03
ES3	0.44	0.65		0.67 ***	0.52 ***	0.34 ***	0.56 ***	0.55 ***	0.51 ***	0.51 ***	0.20 ***	0.40 ***	0.34 ***	0.51 ***	0.52 ***	0.43 ***	0.43 ***	0.43 ***	0.41 ***	-0.13 ***	0.03	0.06 *	0.01	0.01
ES4	0.35	0.52	0.83		0.50 ***	0.38 ***	0.57 ***	0.59 ***	0.56 ***	0.55 ***	0.24 ***	0.43 ***	0.38 ***	0.52 ***	0.53 ***	0.45 ***	0.44 ***	0.44 ***	0.44 ***	-0.13 ***	0.01	0.04	-0.03	-0.04
ES5	0.60	0.73	0.62	0.62		0.57 ***	0.65 ***	0.57 ***	0.51 ***	0.49 ***	0.26 ***	0.38 ***	0.38 ***	0.38 ***	0.38 ***	0.45 ***	0.44 ***	0.47 ***	0.47 ***	0.05	0.02	0.02	0.01	-0.02
ES6	0.45	0.54	0.43	0.49	0.66		0.49 ***	0.40 ***	0.35 ***	0.31 ***	0.23 ***	0.26 ***	0.30 ***	0.25 ***	0.24 ***	0.28 ***	0.27 ***	0.30 ***	0.40 ***	0.12 ***	-0.11 ***	-0.10 ***	-0.11 ***	-0.11 ***
ES7	0.43	0.59	0.71	0.74	0.75	0.57		0.75 ***	0.62 ***	0.61 ***	0.27 ***	0.45 ***	0.41 ***	0.46 ***	0.47 ***	0.53 ***	0.53 ***	0.54 ***	0.49 ***	-0.06 *	0.05	0.05	0.03	-0.01
ES8	0.33	0.49	0.73	0.78	0.65	0.43	0.87		0.67 ***	0.64 ***	0.28 ***	0.47 ***	0.45 ***	0.49 ***	0.51 ***	0.55 ***	0.55 ***	0.54 ***	0.46 ***	-0.13 ***	0.08 **	0.07 *	0.04	0.01
ES9	0.27	0.44	0.70	0.76	0.60	0.40	0.78	0.87		0.79 ***	0.28 ***	0.47 ***	0.43 ***	0.49 ***	0.51 ***	0.61 ***	0.60 ***	0.63 ***	0.46 ***	-0.18 ***	0.07 *	0.10 ***	0.05	0.02
ES10	0.27	0.43	0.71	0.76	0.58	0.36	0.77	0.86	0.92		0.28 ***	0.47 ***	0.42 ***	0.48 ***	0.52 ***	0.64 ***	0.65 ***	0.60 ***	0.43 ***	-0.21 ***	0.07 *	0.10 ***	0.05	0.02
ES11	0.20	0.24	0.27	0.31	0.34	0.28	0.35	0.36	0.37	0.35		0.56 ***	0.49 ***	0.32 ***	0.31 ***	0.35 ***	0.33 ***	0.35 ***	0.33 ***	0.06 *	-0.08 **	-0.07 *	-0.10 ***	-0.10 **
ES12	0.25	0.37	0.55	0.58	0.48	0.33	0.59	0.64	0.64	0.63	0.66		0.65 ***	0.57 ***	0.54 ***	0.51 ***	0.50 ***	0.51 ***	0.43 ***	-0.09 **	0.01	0.01	-0.02	-0.05
ES13	0.27	0.36	0.46	0.51	0.46	0.37	0.52	0.58	0.57	0.55	0.60	0.77		0.51 ***	0.49 ***	0.49 ***	0.47 ***	0.50 ***	0.47 ***	-0.02	0.00	0.01	-0.02	-0.04
ES14	0.27	0.41	0.69	0.70	0.49	0.34	0.62	0.69	0.69	0.68	0.38	0.69	0.62		0.86 ***	0.51 ***	0.49 ***	0.50 ***	0.42 ***	-0.16 ***	0.02	0.04	0.00	-0.04
ES15	0.26	0.38	0.70	0.69	0.48	0.30	0.63	0.71	0.71	0.71	0.36	0.67	0.60	0.95		0.54 ***	0.52 ***	0.52 ***	0.40 ***	-0.18 ***	0.04	0.05	0.01	-0.02
ES16	0.27	0.39	0.60	0.64	0.55	0.35	0.68	0.75	0.79	0.80	0.44	0.66	0.62	0.70	0.72		0.91 ***	0.78 ***	0.50 ***	-0.14 ***	0.04	0.07 *	0.02	-0.01
ES17	0.26	0.39	0.62	0.65	0.55	0.35	0.69	0.77	0.81	0.82	0.42	0.66	0.61	0.70	0.71	0.97		0.77 ***	0.49 ***	-0.15 ***	0.05	0.07 *	0.02	-0.01
ES18	0.31	0.41	0.58	0.61	0.57	0.37	0.67	0.72	0.78	0.75	0.44	0.65	0.63	0.67	0.68	0.90	0.89		0.58 ***	-0.07 *	0.04	0.06	0.02	-0.02
ES19	0.43	0.48	0.53	0.57	0.57	0.50	0.59	0.58	0.58	0.55	0.42	0.55	0.58	0.55	0.52	0.62	0.62	0.70		0.03	-0.06	-0.05	-0.07 *	-0.08 **
ES20	0.13	-0.01	-0.31	-0.35	-0.06	0.04	-0.23	-0.36	-0.40	-0.42	-0.02	-0.24	-0.17	-0.36	-0.37	-0.36	-0.36	-0.28	-0.15		-0.01	-0.05	0.00	-0.02
BQ1	0.00	0.01	0.03	-0.04	0.03	-0.15	0.08	0.11	0.10	0.09	-0.09	0.01	0.01	0.00	0.04	0.05	0.07	0.06	-0.08	0.03		0.63 ***	0.71 ***	0.57 ***
BQ5	-0.03	0.00	0.08	0.02	0.02	-0.11	0.08	0.11	0.13	0.12	-0.09	0.02	0.02	0.03	0.05	0.09	0.10	0.09	-0.05	-0.01	0.75		0.68 ***	0.58 ***
BQ6	-0.04	-0.02	0.00	-0.08	0.00	-0.15	0.04	0.07	0.06	0.05	-0.11	-0.02	-0.02	-0.02	0.01	0.03	0.03	0.03	-0.11	0.04	0.82	0.80		0.63 ***
BQ7	-0.03	-0.03	0.01	-0.09	-0.01	-0.15	0.00	0.03	0.03	0.03	-0.10	-0.05	-0.04	-0.07	-0.02	0.00	0.00	0.00	-0.11	0.02	0.70	0.71	0.77	

Supplementary Table 2. The mean values of the "need for help" ratings of each expression statement (ES1-ES20) in respect to groupings based on the answer values of the background question (BQ), for two groups or three groups.

M_1, M_2 and M_3 show the mean values for each group and the number of persons is denoted by n_1, n_2 and n_3 (n=673). We computed Wilcoxon rank-sum test (i.e., Mann–Whitney U test) between two groups and Kruskal-Wallis test between three groups to identify statistically significant rating differences at significance levels p<0,05, p<0,01 and p<0,001, denoted by symbols *, ** and ***, respectively.

Grouping based on the answer value of the background question	*ES1*	*ES2*	*ES3*	*ES4*	*ES5*	*ES6*	*ES7*	*ES8*	*ES9*	*ES10*	*ES11*	*ES12*	*ES13*	*ES14*	*ES15*	*ES16*	*ES17*	*ES18*	*ES19*	*ES20*
BQ1, two groups: x<7 (n_1=263), x>=7 (n_2=410)	M_1=0.247<br>M_2=0.243	M_1=0.282<br>M_2=0.286	M_1=0.523<br>M_2=0.553	M_1=0.612<br>M_2=0.604	M_1=0.267<br>M_2=0.292	M_1=0.421<br>M_2=0.353 ***	M_1=0.370<br>M_2=0.422 *	M_1=0.497<br>M_2=0.577 **	M_1=0.511<br>M_2=0.594 **	M_1=0.552<br>M_2=0.640 **	M_1=0.464<br>M_2=0.414	M_1=0.465<br>M_2=0.495	M_1=0.440<br>M_2=0.454	M_1=0.605<br>M_2=0.625	M_1=0.584<br>M_2=0.629	M_1=0.460<br>M_2=0.521 *	M_1=0.484<br>M_2=0.560 *	M_1=0.385<br>M_2=0.439 *	M_1=0.396<br>M_2=0.370	M_1=0.257<br>M_2=0.261
BQ1, three groups: x<6 (n_1= 218), 6<=x<8 (n_2=207), x>=8 (n_3=248)	M_1=0.238<br>M_2=0.256<br>M_3=0.241	M_1=0.271<br>M_2=0.307<br>M_3=0.278	M_1=0.522<br>M_2=0.550<br>M_3=0.552	M_1=0.622<br>M_2=0.600<br>M_3=0.601	M_1=0.264<br>M_2=0.312<br>M_3=0.273 *	M_1=0.422<br>M_2=0.383<br>M_3=0.339 **	M_1=0.370<br>M_2=0.414<br>M_3=0.418	M_1=0.493<br>M_2=0.567<br>M_3=0.575 *	M_1=0.506<br>M_2=0.584<br>M_3=0.591 *	M_1=0.551<br>M_2=0.624<br>M_3=0.638	M_1=0.465<br>M_2=0.457<br>M_3=0.386 *	M_1=0.473<br>M_2=0.492<br>M_3=0.485	M_1=0.437<br>M_2=0.463<br>M_3=0.448	M_1=0.616<br>M_2=0.604<br>M_3=0.628	M_1=0.593<br>M_2=0.601<br>M_3=0.635	M_1=0.469<br>M_2=0.514<br>M_3=0.509	M_1=0.488<br>M_2=0.558<br>M_3=0.546	M_1=0.385<br>M_2=0.442<br>M_3=0.426	M_1=0.400<br>M_2=0.392<br>M_3=0.352	M_1=0.246<br>M_2=0.279<br>M_3=0.255
BQ2, two groups: x<2 (n_1=219), x>=2 (n_2=454)	M_1=0.253<br>M_2=0.241	M_1=0.300<br>M_2=0.277	M_1=0.583<br>M_2=0.521 *	M_1=0.622<br>M_2=0.600	M_1=0.288<br>M_2=0.279	M_1=0.337<br>M_2=0.400 **	M_1=0.421<br>M_2=0.392	M_1=0.580<br>M_2=0.529	M_1=0.615<br>M_2=0.536	M_1=0.660<br>M_2=0.579	M_1=0.378<br>M_2=0.461 ***	M_1=0.477<br>M_2=0.487	M_1=0.463<br>M_2=0.442	M_1=0.655<br>M_2=0.599 *	M_1=0.663<br>M_2=0.587 *	M_1=0.534<br>M_2=0.480	M_1=0.573<br>M_2=0.511	M_1=0.448<br>M_2=0.403	M_1=0.369<br>M_2=0.386	M_1=0.233<br>M_2=0.272
BQ4, two groups: x<2 (n_1=364), x>=2 (n_2=309)	M_1=0.249<br>M_2=0.239	M_1=0.290<br>M_2=0.278	M_1=0.549<br>M_2=0.533	M_1=0.615<br>M_2=0.599	M_1=0.285<br>M_2=0.279	M_1=0.354<br>M_2=0.409 **	M_1=0.413<br>M_2=0.388	M_1=0.570<br>M_2=0.517	M_1=0.580<br>M_2=0.540	M_1=0.633<br>M_2=0.574	M_1=0.408<br>M_2=0.464 *	M_1=0.481<br>M_2=0.486	M_1=0.464<br>M_2=0.431	M_1=0.630<br>M_2=0.601	M_1=0.635<br>M_2=0.583	M_1=0.512<br>M_2=0.480	M_1=0.551<br>M_2=0.507	M_1=0.428<br>M_2=0.405	M_1=0.373<br>M_2=0.389	M_1=0.252<br>M_2=0.268
BQ5, two groups: x<7 (n_1=274), x>=7 (n_2=399)	M_1=0.252<br>M_2=0.239	M_1=0.287<br>M_2=0.283	M_1=0.521<br>M_2=0.556	M_1=0.603<br>M_2=0.611	M_1=0.274<br>M_2=0.288	M_1=0.415<br>M_2=0.355 **	M_1=0.378<br>M_2=0.417	M_1=0.510<br>M_2=0.570	M_1=0.515<br>M_2=0.593 **	M_1=0.555<br>M_2=0.640 **	M_1=0.470<br>M_2=0.409 *	M_1=0.477<br>M_2=0.487	M_1=0.441<br>M_2=0.454	M_1=0.598<br>M_2=0.630	M_1=0.584<br>M_2=0.630	M_1=0.459<br>M_2=0.523 *	M_1=0.491<br>M_2=0.558 *	M_1=0.391<br>M_2=0.436	M_1=0.404<br>M_2=0.364	M_1=0.255<br>M_2=0.262
BQ5, three groups: x<6 (n_1=190), 6<=x<8 (n_2=271), x>=8 (n_3=212)	M_1=0.242<br>M_2=0.257<br>M_3=0.232	M_1=0.273<br>M_2=0.296<br>M_3=0.280	M_1=0.496<br>M_2=0.554<br>M_3=0.567	M_1=0.594<br>M_2=0.606<br>M_3=0.621	M_1=0.262<br>M_2=0.304<br>M_3=0.273	M_1=0.412<br>M_2=0.389<br>M_3=0.338 **	M_1=0.362<br>M_2=0.414<br>M_3=0.420	M_1=0.476<br>M_2=0.565<br>M_3=0.583 *	M_1=0.478<br>M_2=0.571<br>M_3=0.624 ***	M_1=0.520<br>M_2=0.614<br>M_3=0.671 ***	M_1=0.461<br>M_2=0.441<br>M_3=0.400	M_1=0.464<br>M_2=0.495<br>M_3=0.486	M_1=0.419<br>M_2=0.472<br>M_3=0.446	M_1=0.589<br>M_2=0.617<br>M_3=0.641	M_1=0.562<br>M_2=0.619<br>M_3=0.646	M_1=0.435<br>M_2=0.517<br>M_3=0.528 *	M_1=0.467<br>M_2=0.546<br>M_3=0.568 *	M_1=0.373<br>M_2=0.431<br>M_3=0.440	M_1=0.402<br>M_2=0.381<br>M_3=0.360	M_1=0.265<br>M_2=0.279<br>M_3=0.230 *
BQ6, two groups: x<7 (n_1=318), x>=7 (n_2=355)	M_1=0.247<br>M_2=0.243	M_1=0.280<br>M_2=0.289	M_1=0.536<br>M_2=0.546	M_1=0.621<br>M_2=0.595	M_1=0.280<br>M_2=0.284	M_1=0.409<br>M_2=0.353 **	M_1=0.387<br>M_2=0.414	M_1=0.522<br>M_2=0.567	M_1=0.541<br>M_2=0.580	M_1=0.592<br>M_2=0.618	M_1=0.478<br>M_2=0.394 ***	M_1=0.489<br>M_2=0.479	M_1=0.448<br>M_2=0.450	M_1=0.620<br>M_2=0.614	M_1=0.609<br>M_2=0.614	M_1=0.486<br>M_2=0.508	M_1=0.519<br>M_2=0.542	M_1=0.412<br>M_2=0.422	M_1=0.403<br>M_2=0.360	M_1=0.250<br>M_2=0.268
BQ6, three groups: x<6 (n_1=240), 6<=x<8 (n_2=229), x>=8 (n_3=204)	M_1=0.256<br>M_2=0.241<br>M_3=0.235	M_1=0.285<br>M_2=0.286<br>M_3=0.282	M_1=0.531<br>M_2=0.549<br>M_3=0.545	M_1=0.628<br>M_2=0.598<br>M_3=0.594	M_1=0.270<br>M_2=0.293<br>M_3=0.284	M_1=0.414<br>M_2=0.374<br>M_3=0.345 *	M_1=0.381<br>M_2=0.401<br>M_3=0.425	M_1=0.506<br>M_2=0.565<br>M_3=0.571	M_1=0.523<br>M_2=0.580<br>M_3=0.586	M_1=0.573<br>M_2=0.616<br>M_3=0.632	M_1=0.470<br>M_2=0.443<br>M_3=0.380 **	M_1=0.483<br>M_2=0.491<br>M_3=0.475	M_1=0.439<br>M_2=0.465<br>M_3=0.443	M_1=0.616<br>M_2=0.611<br>M_3=0.625	M_1=0.595<br>M_2=0.610<br>M_3=0.631	M_1=0.480<br>M_2=0.503<br>M_3=0.511	M_1=0.513<br>M_2=0.540<br>M_3=0.542	M_1=0.403<br>M_2=0.420<br>M_3=0.432	M_1=0.405<br>M_2=0.373<br>M_3=0.360	M_1=0.258<br>M_2=0.251<br>M_3=0.270
BQ7, two groups: x<7 (n_1=201), x>=7 (n_2=472)	M_1=0.262<br>M_2=0.237	M_1=0.300<br>M_2=0.278	M_1=0.550<br>M_2=0.538	M_1=0.657<br>M_2=0.586	M_1=0.287<br>M_2=0.280	M_1=0.434<br>M_2=0.356 ***	M_1=0.405<br>M_2=0.400	M_1=0.536<br>M_2=0.550	M_1=0.541<br>M_2=0.570	M_1=0.590<br>M_2=0.613	M_1=0.484<br>M_2=0.412 **	M_1=0.513<br>M_2=0.471	M_1=0.466<br>M_2=0.442	M_1=0.655<br>M_2=0.601	M_1=0.622<br>M_2=0.607	M_1=0.492<br>M_2=0.500	M_1=0.533<br>M_2=0.530	M_1=0.419<br>M_2=0.417	M_1=0.427<br>M_2=0.360 **	M_1=0.259<br>M_2=0.259
BQ7, three groups: x<6 (n_1=143), 6<=x<8 (n_2=214), x>=8 (n_3=316)	M_1=0.243<br>M_2=0.250<br>M_3=0.241	M_1=0.281<br>M_2=0.291<br>M_3=0.282	M_1=0.520<br>M_2=0.549<br>M_3=0.547	M_1=0.659<br>M_2=0.597<br>M_3=0.591	M_1=0.278<br>M_2=0.292<br>M_3=0.278	M_1=0.432<br>M_2=0.393<br>M_3=0.347 **	M_1=0.400<br>M_2=0.400<br>M_3=0.403	M_1=0.520<br>M_2=0.550<br>M_3=0.554	M_1=0.528<br>M_2=0.567<br>M_3=0.573	M_1=0.589<br>M_2=0.599<br>M_3=0.618	M_1=0.487<br>M_2=0.460<br>M_3=0.392 **	M_1=0.505<br>M_2=0.498<br>M_3=0.464	M_1=0.457<br>M_2=0.459<br>M_3=0.438	M_1=0.648<br>M_2=0.621<br>M_3=0.600	M_1=0.609<br>M_2=0.627<br>M_3=0.602	M_1=0.494<br>M_2=0.503<br>M_3=0.495	M_1=0.536<br>M_2=0.529<br>M_3=0.530	M_1=0.410<br>M_2=0.431<br>M_3=0.411	M_1=0.420<br>M_2=0.382<br>M_3=0.361	M_1=0.248<br>M_2=0.269<br>M_3=0.258
BQ8, two groups: x<2 (n_1=123), x>=2 (n_2=550)	M_1=0.241<br>M_2=0.245	M_1=0.269<br>M_2=0.288	M_1=0.460<br>M_2=0.560 **	M_1=0.516<br>M_2=0.628 ***	M_1=0.284<br>M_2=0.282	M_1=0.390<br>M_2=0.377	M_1=0.359<br>M_2=0.411	M_1=0.474<br>M_2=0.562 *	M_1=0.477<br>M_2=0.580 *	M_1=0.509<br>M_2=0.627 **	M_1=0.367<br>M_2=0.449 **	M_1=0.376<br>M_2=0.507<br>***	M_1=0.389<br>M_2=0.462 *	M_1=0.454<br>M_2=0.653 ***	M_1=0.447<br>M_2=0.648 ***	M_1=0.420<br>M_2=0.515 *	M_1=0.454<br>M_2=0.548 *	M_1=0.355<br>M_2=0.431 *	M_1=0.354<br>M_2=0.386	M_1=0.257<br>M_2=0.260
BQ9, two groups: x<51 (n_1=333), x>=51 (n_2=340)	M_1=0.288<br>M_2=0.202 ***	M_1=0.333<br>M_2=0.237 ***	M_1=0.606<br>M_2=0.479 ***	M_1=0.659<br>M_2=0.557 ***	M_1=0.320<br>M_2=0.245 ***	M_1=0.394<br>M_2=0.365	M_1=0.430<br>M_2=0.373 *	M_1=0.592<br>M_2=0.501 *	M_1=0.598<br>M_2=0.526	M_1=0.655<br>M_2=0.557	M_1=0.410<br>M_2=0.457 *	M_1=0.491<br>M_2=0.476	M_1=0.477<br>M_2=0.422 *	M_1=0.686<br>M_2=0.549 ***	M_1=0.684<br>M_2=0.540 ***	M_1=0.508<br>M_2=0.487	M_1=0.547<br>M_2=0.515	M_1=0.422<br>M_2=0.414	M_1=0.402<br>M_2=0.359 *	M_1=0.241<br>M_2=0.277
BQ9, three groups: x<40 (n_1=225), 40<=x<60 (n_2=231), x>=60 (n_3=217)	M_1=0.301<br>M_2=0.229<br>M_3=0.203 ***	M_1=0.347<br>M_2=0.276<br>M_3=0.229 ***	M_1=0.618<br>M_2=0.553<br>M_3=0.450 ***	M_1=0.655<br>M_2=0.639<br>M_3=0.525 ***	M_1=0.321<br>M_2=0.287<br>M_3=0.236 ***	M_1=0.391<br>M_2=0.388<br>M_3=0.359	M_1=0.434<br>M_2=0.423<br>M_3=0.344 **	M_1=0.597<br>M_2=0.571<br>M_3=0.465 *	M_1=0.606<br>M_2=0.568<br>M_3=0.507	M_1=0.659<br>M_2=0.639<br>M_3=0.515 *	M_1=0.374<br>M_2=0.471<br>M_3=0.456 **	M_1=0.467<br>M_2=0.511<br>M_3=0.471	M_1=0.480<br>M_2=0.437<br>M_3=0.429	M_1=0.691<br>M_2=0.639<br>M_3=0.517 ***	M_1=0.692<br>M_2=0.628<br>M_3=0.510 ***	M_1=0.502<br>M_2=0.506<br>M_3=0.484	M_1=0.545<br>M_2=0.540<br>M_3=0.506	M_1=0.419<br>M_2=0.409<br>M_3=0.425	M_1=0.411<br>M_2=0.378<br>M_3=0.351 *	M_1=0.240<br>M_2=0.231<br>M_3=0.309 *

Supplementary Table 3. The median values of the "need for help" ratings of each expression statement (ES1-ES20) in respect to groupings based on the answer values of the background question (BQ), for two groups or three groups.

Mdn_1, Mdn_2 and Mdn_3 show the median values for each group and the number of persons is denoted by n_1, n_2 and n_3 (n=673).

Grouping based on the answer value of the background question	*ES1*	*ES2*	*ES3*	*ES4*	*ES5*	*ES6*	*ES7*	*ES8*	*ES9*	*ES10*	*ES11*	*ES12*	*ES13*	*ES14*	*ES15*	*ES16*	*ES17*	*ES18*	*ES19*	*ES20*
BQ1, two groups: x<7 (n_1=263), x>=7 (n_2=410)	Mdn_1=0.2<br>Mdn_2=0.2	Mdn_1=0.2<br>Mdn_2=0.3	Mdn_1=0.6<br>Mdn_2=0.7	Mdn_1=0.7<br>Mdn_2=0.7	Mdn_1=0.2<br>Mdn_2=0.3	Mdn_1=0.4<br>Mdn_2=0.3	Mdn_1=0.3<br>Mdn_2=0.5	Mdn_1=0.6<br>Mdn_2=0.7	Mdn_1=0.6<br>Mdn_2=0.8	Mdn_1=0.8<br>Mdn_2=0.8	Mdn_1=0.5<br>Mdn_2=0.4	Mdn_1=0.5<br>Mdn_2=0.6	Mdn_1=0.5<br>Mdn_2=0.5	Mdn_1=0.8<br>Mdn_2=0.8	Mdn_1=0.8<br>Mdn_2=0.8	Mdn_1=0.5<br>Mdn_2=0.6	Mdn_1=0.5<br>Mdn_2=0.7	Mdn_1=0.3<br>Mdn_2=0.5	Mdn_1=0.4<br>Mdn_2=0.4	Mdn_1=0.2<br>Mdn_2=0.1
BQ1, three groups: x<6 (n_1= 218), 6<=x<8 (n_2=207), x>=8 (n_3=248)	Mdn_1=0.2<br>Mdn_2=0.2<br>Mdn_3=0.2	Mdn_1=0.2<br>Mdn_2=0.3<br>Mdn_3=0.25	Mdn_1=0.6<br>Mdn_2=0.7<br>Mdn_3=0.65	Mdn_1=0.7<br>Mdn_2=0.7<br>Mdn_3=0.7	Mdn_1=0.2<br>Mdn_2=0.3<br>Mdn_3=0.2	Mdn_1=0.4<br>Mdn_2=0.4<br>Mdn_3=0.3	Mdn_1=0.3<br>Mdn_2=0.5<br>Mdn_3=0.45	Mdn_1=0.6<br>Mdn_2=0.7<br>Mdn_3=0.7	Mdn_1=0.6<br>Mdn_2=0.8<br>Mdn_3=0.75	Mdn_1=0.7<br>Mdn_2=0.8<br>Mdn_3=0.8	Mdn_1=0.5<br>Mdn_2=0.4<br>Mdn_3=0.3	Mdn_1=0.5<br>Mdn_2=0.6<br>Mdn_3=0.5	Mdn_1=0.4<br>Mdn_2=0.5<br>Mdn_3=0.5	Mdn_1=0.8<br>Mdn_2=0.8<br>Mdn_3=0.8	Mdn_1=0.8<br>Mdn_2=0.8<br>Mdn_3=0.8	Mdn_1=0.5<br>Mdn_2=0.6<br>Mdn_3=0.55	Mdn_1=0.5<br>Mdn_2=0.7<br>Mdn_3=0.6	Mdn_1=0.3<br>Mdn_2=0.5<br>Mdn_3=0.5	Mdn_1=0.4<br>Mdn_2=0.4<br>Mdn_3=0.3	Mdn_1=0.1<br>Mdn_2=0.2<br>Mdn_3=0.1
BQ2, two groups: x<2 (n_1=219), x>=2 (n_2=454)	Mdn_1=0.2<br>Mdn_2=0.2	Mdn_1=0.3<br>Mdn_2=0.2	Mdn_1=0.7<br>Mdn_2=0.6	Mdn_1=0.7<br>Mdn_2=0.7	Mdn_1=0.3<br>Mdn_2=0.2	Mdn_1=0.3<br>Mdn_2=0.4	Mdn_1=0.4<br>Mdn_2=0.4	Mdn_1=0.7<br>Mdn_2=0.7	Mdn_1=0.8<br>Mdn_2=0.7	Mdn_1=0.8<br>Mdn_2=0.8	Mdn_1=0.3<br>Mdn_2=0.5	Mdn_1=0.5<br>Mdn_2=0.5	Mdn_1=0.5<br>Mdn_2=0.5	Mdn_1=0.8<br>Mdn_2=0.8	Mdn_1=0.8<br>Mdn_2=0.8	Mdn_1=0.6<br>Mdn_2=0.5	Mdn_1=0.7<br>Mdn_2=0.6	Mdn_1=0.5<br>Mdn_2=0.4	Mdn_1=0.4<br>Mdn_2=0.4	Mdn_1=0.1<br>Mdn_2=0.2
BQ4, two groups: x<2 (n_1=364), x>=2 (n_2=309)	Mdn_1=0.2<br>Mdn_2=0.2	Mdn_1=0.3<br>Mdn_2=0.2	Mdn_1=0.65<br>Mdn_2=0.6	Mdn_1=0.7<br>Mdn_2=0.7	Mdn_1=0.3<br>Mdn_2=0.2	Mdn_1=0.3<br>Mdn_2=0.4	Mdn_1=0.4<br>Mdn_2=0.4	Mdn_1=0.7<br>Mdn_2=0.7	Mdn_1=0.7<br>Mdn_2=0.7	Mdn_1=0.8<br>Mdn_2=0.8	Mdn_1=0.35<br>Mdn_2=0.5	Mdn_1=0.5<br>Mdn_2=0.6	Mdn_1=0.5<br>Mdn_2=0.4	Mdn_1=0.8<br>Mdn_2=0.8	Mdn_1=0.8<br>Mdn_2=0.8	Mdn_1=0.6<br>Mdn_2=0.5	Mdn_1=0.6<br>Mdn_2=0.6	Mdn_1=0.45<br>Mdn_2=0.4	Mdn_1=0.4<br>Mdn_2=0.4	Mdn_1=0.1<br>Mdn_2=0.2
BQ5, two groups: x<7 (n_1=274), x>=7 (n_2=399)	Mdn_1=0.2<br>Mdn_2=0.2	Mdn_1=0.2<br>Mdn_2=0.3	Mdn_1=0.6<br>Mdn_2=0.7	Mdn_1=0.7<br>Mdn_2=0.7	Mdn_1=0.2<br>Mdn_2=0.3	Mdn_1=0.4<br>Mdn_2=0.3	Mdn_1=0.3<br>Mdn_2=0.4	Mdn_1=0.6<br>Mdn_2=0.7	Mdn_1=0.7<br>Mdn_2=0.8	Mdn_1=0.8<br>Mdn_2=0.8	Mdn_1=0.5<br>Mdn_2=0.4	Mdn_1=0.5<br>Mdn_2=0.5	Mdn_1=0.45<br>Mdn_2=0.5	Mdn_1=0.8<br>Mdn_2=0.8	Mdn_1=0.75<br>Mdn_2=0.8	Mdn_1=0.5<br>Mdn_2=0.6	Mdn_1=0.6<br>Mdn_2=0.7	Mdn_1=0.4<br>Mdn_2=0.5	Mdn_1=0.4<br>Mdn_2=0.3	Mdn_1=0.2<br>Mdn_2=0.1
BQ5, three groups: x<6 (n_1=190), 6<=x<8 (n_2=271), x>=8 (n_3=212)	Mdn_1=0.2<br>Mdn_2=0.2<br>Mdn_3=0.2	Mdn_1=0.2<br>Mdn_2=0.3<br>Mdn_3=0.2	Mdn_1=0.55<br>Mdn_2=0.7<br>Mdn_3=0.7	Mdn_1=0.7<br>Mdn_2=0.7<br>Mdn_3=0.7	Mdn_1=0.2<br>Mdn_2=0.3<br>Mdn_3=0.2	Mdn_1=0.4<br>Mdn_2=0.4<br>Mdn_3=0.3	Mdn_1=0.3<br>Mdn_2=0.5<br>Mdn_3=0.4	Mdn_1=0.6<br>Mdn_2=0.8<br>Mdn_3=0.7	Mdn_1=0.55<br>Mdn_2=0.8<br>Mdn_3=0.8	Mdn_1=0.7<br>Mdn_2=0.8<br>Mdn_3=0.8	Mdn_1=0.5<br>Mdn_2=0.4<br>Mdn_3=0.3	Mdn_1=0.5<br>Mdn_2=0.5<br>Mdn_3=0.5	Mdn_1=0.4<br>Mdn_2=0.5<br>Mdn_3=0.45	Mdn_1=0.8<br>Mdn_2=0.8<br>Mdn_3=0.8	Mdn_1=0.7<br>Mdn_2=0.8<br>Mdn_3=0.9	Mdn_1=0.5<br>Mdn_2=0.6<br>Mdn_3=0.6	Mdn_1=0.5<br>Mdn_2=0.6<br>Mdn_3=0.7	Mdn_1=0.3<br>Mdn_2=0.5<br>Mdn_3=0.5	Mdn_1=0.4<br>Mdn_2=0.4<br>Mdn_3=0.3	Mdn_1=0.2<br>Mdn_2=0.2<br>Mdn_3=0.1
BQ6, two groups: x<7 (n_1=318), x>=7 (n_2=355)	Mdn_1=0.2<br>Mdn_2=0.2	Mdn_1=0.2<br>Mdn_2=0.3	Mdn_1=0.6<br>Mdn_2=0.7	Mdn_1=0.7<br>Mdn_2=0.7	Mdn_1=0.2<br>Mdn_2=0.3	Mdn_1=0.4<br>Mdn_2=0.3	Mdn_1=0.4<br>Mdn_2=0.4	Mdn_1=0.7<br>Mdn_2=0.7	Mdn_1=0.7<br>Mdn_2=0.7	Mdn_1=0.8<br>Mdn_2=0.8	Mdn_1=0.5<br>Mdn_2=0.3	Mdn_1=0.55<br>Mdn_2=0.5	Mdn_1=0.5<br>Mdn_2=0.5	Mdn_1=0.8<br>Mdn_2=0.8	Mdn_1=0.8<br>Mdn_2=0.8	Mdn_1=0.5<br>Mdn_2=0.6	Mdn_1=0.6<br>Mdn_2=0.6	Mdn_1=0.4<br>Mdn_2=0.5	Mdn_1=0.4<br>Mdn_2=0.3	Mdn_1=0.2<br>Mdn_2=0.2
BQ6, three groups: x<6 (n_1=240), 6<=x<8 (n_2=229), x>=8 (n_3=204)	Mdn_1=0.2<br>Mdn_2=0.2<br>Mdn_3=0.2	Mdn_1=0.2<br>Mdn_2=0.3<br>Mdn_3=0.2	Mdn_1=0.6<br>Mdn_2=0.6<br>Mdn_3=0.65	Mdn_1=0.7<br>Mdn_2=0.7<br>Mdn_3=0.7	Mdn_1=0.2<br>Mdn_2=0.3<br>Mdn_3=0.3	Mdn_1=0.4<br>Mdn_2=0.4<br>Mdn_3=0.3	Mdn_1=0.4<br>Mdn_2=0.4<br>Mdn_3=0.5	Mdn_1=0.6<br>Mdn_2=0.7<br>Mdn_3=0.7	Mdn_1=0.6<br>Mdn_2=0.8<br>Mdn_3=0.7	Mdn_1=0.8<br>Mdn_2=0.8<br>Mdn_3=0.8	Mdn_1=0.5<br>Mdn_2=0.4<br>Mdn_3=0.3	Mdn_1=0.5<br>Mdn_2=0.5<br>Mdn_3=0.5	Mdn_1=0.4<br>Mdn_2=0.5<br>Mdn_3=0.5	Mdn_1=0.8<br>Mdn_2=0.8<br>Mdn_3=0.8	Mdn_1=0.8<br>Mdn_2=0.8<br>Mdn_3=0.8	Mdn_1=0.5<br>Mdn_2=0.5<br>Mdn_3=0.6	Mdn_1=0.6<br>Mdn_2=0.7<br>Mdn_3=0.6	Mdn_1=0.4<br>Mdn_2=0.4<br>Mdn_3=0.5	Mdn_1=0.4<br>Mdn_2=0.4<br>Mdn_3=0.3	Mdn_1=0.2<br>Mdn_2=0.1<br>Mdn_3=0.1
BQ7, two groups: x<7 (n_1=201), x>=7 (n_2=472)	Mdn_1=0.2<br>Mdn_2=0.2	Mdn_1=0.3<br>Mdn_2=0.2	Mdn_1=0.6<br>Mdn_2=0.6	Mdn_1=0.7<br>Mdn_2=0.7	Mdn_1=0.3<br>Mdn_2=0.3	Mdn_1=0.4<br>Mdn_2=0.3	Mdn_1=0.4<br>Mdn_2=0.4	Mdn_1=0.7<br>Mdn_2=0.7	Mdn_1=0.7<br>Mdn_2=0.7	Mdn_1=0.8<br>Mdn_2=0.8	Mdn_1=0.5<br>Mdn_2=0.4	Mdn_1=0.6<br>Mdn_2=0.5	Mdn_1=0.5<br>Mdn_2=0.5	Mdn_1=0.8<br>Mdn_2=0.8	Mdn_1=0.8<br>Mdn_2=0.8	Mdn_1=0.6<br>Mdn_2=0.5	Mdn_1=0.6<br>Mdn_2=0.6	Mdn_1=0.4<br>Mdn_2=0.4	Mdn_1=0.4<br>Mdn_2=0.35	Mdn_1=0.2<br>Mdn_2=0.1
BQ7, three groups: x<6 (n_1=143), 6<=x<8 (n_2=214), x>=8 (n_3=316)	Mdn_1=0.2<br>Mdn_2=0.2<br>Mdn_3=0.2	Mdn_1=0.2<br>Mdn_2=0.3<br>Mdn_3=0.2	Mdn_1=0.6<br>Mdn_2=0.65<br>Mdn_3=0.65	Mdn_1=0.7<br>Mdn_2=0.7<br>Mdn_3=0.7	Mdn_1=0.2<br>Mdn_2=0.3<br>Mdn_3=0.25	Mdn_1=0.4<br>Mdn_2=0.4<br>Mdn_3=0.3	Mdn_1=0.4<br>Mdn_2=0.5<br>Mdn_3=0.4	Mdn_1=0.6<br>Mdn_2=0.7<br>Mdn_3=0.7	Mdn_1=0.6<br>Mdn_2=0.8<br>Mdn_3=0.7	Mdn_1=0.8<br>Mdn_2=0.8<br>Mdn_3=0.8	Mdn_1=0.5<br>Mdn_2=0.5<br>Mdn_3=0.3	Mdn_1=0.6<br>Mdn_2=0.6<br>Mdn_3=0.5	Mdn_1=0.5<br>Mdn_2=0.5<br>Mdn_3=0.45	Mdn_1=0.8<br>Mdn_2=0.8<br>Mdn_3=0.8	Mdn_1=0.8<br>Mdn_2=0.8<br>Mdn_3=0.8	Mdn_1=0.6<br>Mdn_2=0.6<br>Mdn_3=0.5	Mdn_1=0.6<br>Mdn_2=0.6<br>Mdn_3=0.6	Mdn_1=0.4<br>Mdn_2=0.5<br>Mdn_3=0.4	Mdn_1=0.4<br>Mdn_2=0.4<br>Mdn_3=0.4	Mdn_1=0.2<br>Mdn_2=0.2<br>Mdn_3=0.1
BQ8, two groups: x<2 (n_1=123), x>=2 (n_2=550)	Mdn_1=0.2<br>Mdn_2=0.2	Mdn_1=0.2<br>Mdn_2=0.25	Mdn_1=0.5<br>Mdn_2=0.7	Mdn_1=0.6<br>Mdn_2=0.7	Mdn_1=0.2<br>Mdn_2=0.3	Mdn_1=0.4<br>Mdn_2=0.4	Mdn_1=0.3<br>Mdn_2=0.4	Mdn_1=0.6<br>Mdn_2=0.7	Mdn_1=0.5<br>Mdn_2=0.7	Mdn_1=0.6<br>Mdn_2=0.8	Mdn_1=0.3<br>Mdn_2=0.4	Mdn_1=0.3<br>Mdn_2=0.6	Mdn_1=0.4<br>Mdn_2=0.5	Mdn_1=0.5<br>Mdn_2=0.8	Mdn_1=0.5<br>Mdn_2=0.8	Mdn_1=0.5<br>Mdn_2=0.6	Mdn_1=0.5<br>Mdn_2=0.65	Mdn_1=0.3<br>Mdn_2=0.5	Mdn_1=0.3<br>Mdn_2=0.4	Mdn_1=0.1<br>Mdn_2=0.2
BQ9, two groups: x<51 (n_1=333), x>=51 (n_2=340)	Mdn_1=0.2<br>Mdn2=0.2	Mdn_1=0.3<br>Mdn_2=0.2	Mdn_1=0.7<br>Mdn_2=0.5	Mdn_1=0.7<br>Mdn_2=0.7	Mdn_1=0.3<br>Mdn_2=0.2	Mdn_1=0.4<br>Mdn_2=0.3	Mdn_1=0.5<br>Mdn_2=0.4	Mdn_1=0.7<br>Mdn_2=0.6	Mdn_1=0.7<br>Mdn_2=0.7	Mdn_1=0.8<br>Mdn_2=0.8	Mdn_1=0.4<br>Mdn_2=0.5	Mdn_1=0.5<br>Mdn_2=0.5	Mdn_1=0.5<br>Mdn_2=0.4	Mdn_1=0.8<br>Mdn_2=0.7	Mdn_1=0.8<br>Mdn_2=0.7	Mdn_1=0.6<br>Mdn_2=0.5	Mdn_1=0.6<br>Mdn_2=0.6	Mdn_1=0.4<br>Mdn_2=0.4	Mdn_1=0.4<br>Mdn_2=0.3	Mdn_1=0.2<br>Mdn_2=0.15
BQ9, three groups: x<40 (n_1=225), 40<=x<60 (n_2=231), x>=60 (n_3=217)	Mdn_1=0.3<br>Mdn_2=0.2<br>Mdn_3=0.1	Mdn_1=0.3<br>Mdn_2=0.2<br>Mdn_3=0.2	Mdn_1=0.7<br>Mdn_2=0.7<br>Mdn_3=0.5	Mdn_1=0.7<br>Mdn_2=0.8<br>Mdn_3=0.6	Mdn_1=0.3<br>Mdn_2=0.2<br>Mdn_3=0.2	Mdn_1=0.4<br>Mdn_2=0.4<br>Mdn_3=0.3	Mdn_1=0.5<br>Mdn_2=0.4<br>Mdn_3=0.3	Mdn_1=0.7<br>Mdn_2=0.7<br>Mdn_3=0.6	Mdn_1=0.7<br>Mdn_2=0.7<br>Mdn_3=0.7	Mdn_1=0.8<br>Mdn_2=0.8<br>Mdn_3=0.7	Mdn_1=0.3<br>Mdn_2=0.5<br>Mdn_3=0.5	Mdn_1=0.5<br>Mdn_2=0.6<br>Mdn_3=0.5	Mdn_1=0.5<br>Mdn_2=0.4<br>Mdn_3=0.4	Mdn_1=0.8<br>Mdn_2=0.8<br>Mdn_3=0.5	Mdn_1=0.8<br>Mdn_2=0.8<br>Mdn_3=0.5	Mdn_1=0.5<br>Mdn_2=0.6<br>Mdn_3=0.6	Mdn_1=0.6<br>Mdn_2=0.6<br>Mdn_3=0.6	Mdn_1=0.4<br>Mdn_2=0.4<br>Mdn_3=0.5	Mdn_1=0.4<br>Mdn_2=0.4<br>Mdn_3=0.3	Mdn_1=0.2<br>Mdn_2=0.1<br>Mdn_3=0.2

Supplementary Table 4. The standard deviation values of the "need for help" ratings of each expression statement (ES1-ES20) in respect to groupings based on the answer values of the background question (BQ), for two groups or three groups.

SD_1, SD_2 and SD_3 show the standard deviation values for each group and the number of persons is denoted by n_1, n_2 and n_3 (n=673).

Grouping based on the answer value of the background question	*ES1*	*ES2*	*ES3*	*ES4*	*ES5*	*ES6*	*ES7*	*ES8*	*ES9*	*ES10*	*ES11*	*ES12*	*ES13*	*ES14*	*ES15*	*ES16*	*ES17*	*ES18*	*ES19*	*ES20*
BQ1, two groups: x<7 (n_1=263), x>=7 (n_2=410)	SD_1=0.22<br>SD_2=0.21	SD_1=0.23<br>SD_2=0.22	SD_1=0.32<br>SD_2=0.34	SD_1=0.29<br>SD_2=0.34	SD_1=0.24<br>SD_2=0.24	SD_1=0.26<br>SD_2=0.24	SD_1=0.31<br>SD_2=0.30	SD_1=0.37<br>SD_2=0.36	SD_1=0.39<br>SD_2=0.38	SD_1=0.42<br>SD_2=0.41	SD_1=0.32<br>SD_2=0.30	SD_1=0.35<br>SD_2=0.34	SD_1=0.32<br>SD_2=0.32	SD_1=0.37<br>SD_2=0.38	SD_1=0.39<br>SD_2=0.40	SD_1=0.37<br>SD_2=0.36	SD_1=0.38<br>SD_2=0.37	SD_1=0.32<br>SD_2=0.31	SD_1=0.27<br>SD_2=0.27	SD_1=0.27<br>SD_2=0.30
BQ1, three groups: x<6 (n_1= 218), 6<=x<8 (n_2=207), x>=8 (n_3=248)	SD_1=0.22<br>SD_2=0.21<br>SD_3=0.21	SD_1=0.23<br>SD_2=0.23<br>SD_3=0.22	SD_1=0.32<br>SD_2=0.33<br>SD_3=0.34	SD_1=0.29<br>SD_2=0.32<br>SD_3=0.36	SD_1=0.25<br>SD_2=0.24<br>SD_3=0.23	SD_1=0.26<br>SD_2=0.24<br>SD_3=0.23	SD_1=0.31<br>SD_2=0.30<br>SD_3=0.30	SD_1=0.37<br>SD_2=0.36<br>SD_3=0.36	SD_1=0.39<br>SD_2=0.39<br>SD_3=0.38	SD_1=0.42<br>SD_2=0.42<br>SD_3=0.40	SD_1=0.32<br>SD_2=0.30<br>SD_3=0.30	SD_1=0.35<br>SD_2=0.33<br>SD_3=0.34	SD_1=0.33<br>SD_2=0.31<br>SD_3=0.32	SD_1=0.37<br>SD_2=0.37<br>SD_3=0.39	SD_1=0.39<br>SD_2=0.39<br>SD_3=0.40	SD_1=0.38<br>SD_2=0.36<br>SD_3=0.36	SD_1=0.39<br>SD_2=0.38<br>SD_3=0.37	SD_1=0.32<br>SD_2=0.32<br>SD_3=0.31	SD_1=0.28<br>SD_2=0.26<br>SD_3=0.27	SD_1=0.27<br>SD_2=0.29<br>SD_3=0.30
BQ2, two groups: x<2 (n_1=219), x>=2 (n_2=454)	SD_1=0.21<br>SD_2=0.22	SD_1=0.22<br>SD_2=0.23	SD_1=0.31<br>SD_2=0.34	SD_1=0.32<br>SD_2=0.32	SD_1=0.21<br>SD_2=0.25	SD_1=0.22<br>SD_2=0.26	SD_1=0.28<br>SD_2=0.32	SD_1=0.34<br>SD_2=0.38	SD_1=0.36<br>SD_2=0.40	SD_1=0.38<br>SD_2=0.43	SD_1=0.29<br>SD_2=0.31	SD_1=0.33<br>SD_2=0.35	SD_1=0.31<br>SD_2=0.33	SD_1=0.38<br>SD_2=0.38	SD_1=0.38<br>SD_2=0.40	SD_1=0.34<br>SD_2=0.38	SD_1=0.35<br>SD_2=0.39	SD_1=0.30<br>SD_2=0.33	SD_1=0.26<br>SD_2=0.28	SD_1=0.27<br>SD_2=0.29
BQ4, two groups: x<2 (n_1=364), x>=2 (n_2=309)	SD_1=0.21<br>SD_2=0.22	SD_1=0.22<br>SD_2=0.23	SD_1=0.33<br>SD_2=0.33	SD_1=0.32<br>SD_2=0.33	SD_1=0.23<br>SD_2=0.25	SD_1=0.23<br>SD_2=0.26	SD_1=0.30<br>SD_2=0.32	SD_1=0.35<br>SD_2=0.38	SD_1=0.38<br>SD_2=0.40	SD_1=0.40<br>SD_2=0.43	SD_1=0.31<br>SD_2=0.31	SD_1=0.34<br>SD_2=0.35	SD_1=0.32<br>SD_2=0.32	SD_1=0.38<br>SD_2=0.38	SD_1=0.39<br>SD_2=0.40	SD_1=0.36<br>SD_2=0.38	SD_1=0.37<br>SD_2=0.39	SD_1=0.31<br>SD_2=0.33	SD_1=0.26<br>SD_2=0.28	SD_1=0.29<br>SD_2=0.29
BQ5, two groups: x<7 (n_1=274), x>=7 (n_2=399)	SD_1=0.23<br>SD_2=0.21	SD_1=0.23<br>SD_2=0.22	SD_1=0.32<br>SD_2=0.34	SD_1=0.30<br>SD_2=0.34	SD_1=0.24<br>SD_2=0.24	SD_1=0.26<br>SD_2=0.24	SD_1=0.31<br>SD_2=0.30	SD_1=0.37<br>SD_2=0.36	SD_1=0.39<br>SD_2=0.38	SD_1=0.42<br>SD_2=0.41	SD_1=0.32<br>SD_2=0.30	SD_1=0.35<br>SD_2=0.34	SD_1=0.33<br>SD_2=0.32	SD_1=0.37<br>SD_2=0.38	SD_1=0.39<br>SD_2=0.40	SD_1=0.37<br>SD_2=0.36	SD_1=0.38<br>SD_2=0.37	SD_1=0.32<br>SD_2=0.31	SD_1=0.27<br>SD_2=0.27	SD_1=0.26<br>SD_2=0.30
BQ5, three groups: x<6 (n_1=190), 6<=x<8 (n_2=271), x>=8 (n_3=212)	SD_1=0.24<br>SD_2=0.21<br>SD_3=0.21	SD_1=0.24<br>SD_2=0.22<br>SD_3=0.22	SD_1=0.33<br>SD_2=0.32<br>SD_3=0.34	SD_1=0.30<br>SD_2=0.32<br>SD_3=0.34	SD_1=0.25<br>SD_2=0.24<br>SD_3=0.23	SD_1=0.26<br>SD_2=0.24<br>SD_3=0.23	SD_1=0.32<br>SD_2=0.30<br>SD_3=0.30	SD_1=0.38<br>SD_2=0.37<br>SD_3=0.35	SD_1=0.40<br>SD_2=0.39<br>SD_3=0.37	SD_1=0.42<br>SD_2=0.42<br>SD_3=0.39	SD_1=0.32<br>SD_2=0.31<br>SD_3=0.30	SD_1=0.36<br>SD_2=0.33<br>SD_3=0.34	SD_1=0.33<br>SD_2=0.32<br>SD_3=0.31	SD_1=0.38<br>SD_2=0.37<br>SD_3=0.39	SD_1=0.40<br>SD_2=0.39<br>SD_3=0.40	SD_1=0.38<br>SD_2=0.36<br>SD_3=0.36	SD_1=0.39<br>SD_2=0.37<br>SD_3=0.37	SD_1=0.33<br>SD_2=0.31<br>SD_3=0.31	SD_1=0.28<br>SD_2=0.27<br>SD_3=0.26	SD_1=0.26<br>SD_2=0.29<br>SD_3=0.30
BQ6, two groups: x<7 (n_1=318), x>=7 (n_2=355)	SD_1=0.22<br>SD_2=0.21	SD_1=0.23<br>SD_2=0.22	SD_1=0.32<br>SD_2=0.34	SD_1=0.30<br>SD_2=0.34	SD_1=0.25<br>SD_2=0.23	SD_1=0.25<br>SD_2=0.24	SD_1=0.31<br>SD_2=0.30	SD_1=0.37<br>SD_2=0.36	SD_1=0.39<br>SD_2=0.39	SD_1=0.42<br>SD_2=0.41	SD_1=0.32<br>SD_2=0.30	SD_1=0.35<br>SD_2=0.33	SD_1=0.33<br>SD_2=0.32	SD_1=0.37<br>SD_2=0.39	SD_1=0.39<br>SD_2=0.40	SD_1=0.37<br>SD_2=0.37	SD_1=0.38<br>SD_2=0.38	SD_1=0.32<br>SD_2=0.32	SD_1=0.27<br>SD_2=0.27	SD_1=0.27<br>SD_2=0.30
BQ6, three groups: x<6 (n_1=240), 6<=x<8 (n_2=229), x>=8 (n_3=204)	SD_1=0.23<br>SD_2=0.21<br>SD_3=0.21	SD_1=0.23<br>SD_2=0.23<br>SD_3=0.22	SD_1=0.32<br>SD_2=0.33<br>SD_3=0.34	SD_1=0.29<br>SD_2=0.32<br>SD_3=0.36	SD_1=0.24<br>SD_2=0.24<br>SD_3=0.23	SD_1=0.26<br>SD_2=0.23<br>SD_3=0.24	SD_1=0.31<br>SD_2=0.30<br>SD_3=0.31	SD_1=0.37<br>SD_2=0.36<br>SD_3=0.36	SD_1=0.39<br>SD_2=0.38<br>SD_3=0.39	SD_1=0.42<br>SD_2=0.41<br>SD_3=0.41	SD_1=0.31<br>SD_2=0.31<br>SD_3=0.30	SD_1=0.36<br>SD_2=0.33<br>SD_3=0.34	SD_1=0.33<br>SD_2=0.32<br>SD_3=0.32	SD_1=0.37<br>SD_2=0.38<br>SD_3=0.39	SD_1=0.39<br>SD_2=0.39<br>SD_3=0.40	SD_1=0.38<br>SD_2=0.36<br>SD_3=0.37	SD_1=0.39<br>SD_2=0.36<br>SD_3=0.38	SD_1=0.33<br>SD_2=0.31<br>SD_3=0.32	SD_1=0.27<br>SD_2=0.26<br>SD_3=0.28	SD_1=0.27<br>SD_2=0.29<br>SD_3=0.31
BQ7, two groups: x<7 (n_1=201), x>=7 (n_2=472)	SD_1=0.22<br>SD_2=0.21	SD_1=0.23<br>SD_2=0.23	SD_1=0.31<br>SD_2=0.34	SD_1=0.28<br>SD_2=0.34	SD_1=0.24<br>SD_2=0.24	SD_1=0.26<br>SD_2=0.24	SD_1=0.31<br>SD_2=0.31	SD_1=0.36<br>SD_2=0.37	SD_1=0.38<br>SD_2=0.39	SD_1=0.41<br>SD_2=0.42	SD_1=0.31<br>SD_2=0.31	SD_1=0.35<br>SD_2=0.34	SD_1=0.32<br>SD_2=0.32	SD_1=0.35<br>SD_2=0.39	SD_1=0.38<br>SD_2=0.40	SD_1=0.37<br>SD_2=0.37	SD_1=0.38<br>SD_2=0.38	SD_1=0.32<br>SD_2=0.32	SD_1=0.27<br>SD_2=0.27	SD_1=0.27<br>SD_2=0.30
BQ7, three groups: x<6 (n_1=143), 6<=x<8 (n_2=214), x>=8 (n_3=316)	SD_1=0.22<br>SD_2=0.22<br>SD_3=0.21	SD_1=0.23<br>SD_2=0.22<br>SD_3=0.23	SD_1=0.32<br>SD_2=0.31<br>SD_3=0.35	SD_1=0.27<br>SD_2=0.32<br>SD_3=0.35	SD_1=0.24<br>SD_2=0.25<br>SD_3=0.23	SD_1=0.26<br>SD_2=0.25<br>SD_3=0.23	SD_1=0.31<br>SD_2=0.31<br>SD_3=0.31	SD_1=0.36<br>SD_2=0.37<br>SD_3=0.36	SD_1=0.38<br>SD_2=0.39<br>SD_3=0.39	SD_1=0.42<br>SD_2=0.41<br>SD_3=0.42	SD_1=0.32<br>SD_2=0.31<br>SD_3=0.30	SD_1=0.35<br>SD_2=0.33<br>SD_3=0.34	SD_1=0.32<br>SD_2=0.32<br>SD_3=0.32	SD_1=0.35<br>SD_2=0.37<br>SD_3=0.40	SD_1=0.38<br>SD_2=0.38<br>SD_3=0.41	SD_1=0.37<br>SD_2=0.38<br>SD_3=0.36	SD_1=0.38<br>SD_2=0.38<br>SD_3=0.37	SD_1=0.31<br>SD_2=0.33<br>SD_3=0.32	SD_1=0.26<br>SD_2=0.27<br>SD_3=0.27	SD_1=0.26<br>SD_2=0.29<br>SD_3=0.30
BQ8, two groups: x<2 (n_1=123), x>=2 (n_2=550)	SD_1=0.23<br>SD_2=0.21	SD_1=0.25<br>SD_2=0.22	SD_1=0.33<br>SD_2=0.33	SD_1=0.32<br>SD_2=0.32	SD_1=0.26<br>SD_2=0.23	SD_1=0.26<br>SD_2=0.24	SD_1=0.31<br>SD_2=0.30	SD_1=0.38<br>SD_2=0.36	SD_1=0.40<br>SD_2=0.38	SD_1=0.42<br>SD_2=0.41	SD_1=0.32<br>SD_2=0.30	SD_1=0.34<br>SD_2=0.34	SD_1=0.32<br>SD_2=0.32	SD_1=0.38<br>SD_2=0.37	SD_1=0.39<br>SD_2=0.39	SD_1=0.38<br>SD_2=0.36	SD_1=0.40<br>SD_2=0.37	SD_1=0.33<br>SD_2=0.31	SD_1=0.28<br>SD_2=0.27	SD_1=0.29<br>SD_2=0.29
BQ9, two groups: x<51 (n_1=333), x>=51 (n_2=340)	SD_1=0.22<br>SD_2=0.20	SD_1=0.22<br>SD_2=0.22	SD_1=0.31<br>SD_2=0.35	SD_1=0.29<br>SD_2=0.34	SD_1=0.23<br>SD_2=0.24	SD_1=0.24<br>SD_2=0.26	SD_1=0.28<br>SD_2=0.33	SD_1=0.33<br>SD_2=0.39	SD_1=0.36<br>SD_2=0.41	SD_1=0.38<br>SD_2=0.44	SD_1=0.30<br>SD_2=0.31	SD_1=0.32<br>SD_2=0.36	SD_1=0.31<br>SD_2=0.33	SD_1=0.35<br>SD_2=0.40	SD_1=0.36<br>SD_2=0.41	SD_1=0.33<br>SD_2=0.40	SD_1=0.34<br>SD_2=0.41	SD_1=0.29<br>SD_2=0.35	SD_1=0.25<br>SD_2=0.29	SD_1=0.27<br>SD_2=0.30
BQ9, three groups: x<40 (n_1=225), 40<=x<60 (n_2=231), x>=60 (n_3=217)	SD_1=0.22<br>SD_2=0.21<br>SD_3=0.21	SD_1=0.22<br>SD_2=0.22<br>SD_3=0.22	SD_1=0.29<br>SD_2=0.34<br>SD_3=0.34	SD_1=0.28<br>SD_2=0.33<br>SD_3=0.35	SD_1=0.22<br>SD_2=0.24<br>SD_3=0.25	SD_1=0.24<br>SD_2=0.25<br>SD_3=0.25	SD_1=0.28<br>SD_2=0.31<br>SD_3=0.33	SD_1=0.33<br>SD_2=0.36<br>SD_3=0.39	SD_1=0.35<br>SD_2=0.38<br>SD_3=0.43	SD_1=0.37<br>SD_2=0.40<br>SD_3=0.45	SD_1=0.29<br>SD_2=0.32<br>SD_3=0.31	SD_1=0.31<br>SD_2=0.35<br>SD_3=0.37	SD_1=0.30<br>SD_2=0.32<br>SD_3=0.34	SD_1=0.33<br>SD_2=0.38<br>SD_3=0.40	SD_1=0.35<br>SD_2=0.40<br>SD_3=0.42	SD_1=0.32<br>SD_2=0.36<br>SD_3=0.42	SD_1=0.33<br>SD_2=0.37<br>SD_3=0.43	SD_1=0.28<br>SD_2=0.31<br>SD_3=0.37	SD_1=0.24<br>SD_2=0.26<br>SD_3=0.30	SD_1=0.26<br>SD_2=0.28<br>SD_3=0.32

Supplementary Table 5. Results of Wilcoxon rank-sum test (i.e., Mann–Whitney U test) between two groups and Kruskal-Wallis test between three groups to identify statistically significant rating differences for expression statements ES1-ES20 in respect to groupings based on the answer values of each background question (BQ).
This table shows the p-values concerning the rating differences between groups when the statistical significance levels were defined as p<0.05, p<0.01 and p<0.001, denoted by symbols *, ** and ***, respectively (n=673).

Grouping based on the answer value of the background question	*ES1*	*ES2*	*ES3*	*ES4*	*ES5*	*ES6*	*ES7*	*ES8*	*ES9*	*ES10*	*ES11*	*ES12*	*ES13*	*ES14*	*ES15*	*ES16*	*ES17*	*ES18*	*ES19*	*ES20*
BQ1, two groups: x<7 (n_1=263), x>=7 (n_2=410)	0.9197	0.6643	0.1491	0.4769	0.1094	0.001105 **	0.03835 *	0.007304 **	0.006771 **	0.004866 **	0.05642	0.3291	0.6438	0.3987	0.1316	0.04026 *	0.01429 *	0.0358 *	0.3167	0.5732
BQ1, three groups: x<6 (n_1= 218), 6<=x<8 (n_2=207), x>=8 (n_3=248)	0.331	0.1774	0.4204	0.815	0.04487 *	0.002876 **	0.2161	0.04889 *	0.03546 *	0.05269	0.01077 *	0.9052	0.7382	0.4413	0.398	0.4829	0.2057	0.168	0.1383	0.3959
BQ2, two groups: x<2 (n_1=219), x>=2 (n_2=454)	0.2048	0.1175	0.04755 *	0.4151	0.2517	0.003857 **	0.269	0.2825	0.05099	0.0657	0.001373 **	0.6126	0.4365	0.04 *	0.01885 *	0.122	0.1097	0.09556	0.5401	0.1131
BQ4, two groups: x<2 (n_1=364), x>=2 (n_2=309)	0.3973	0.3349	0.5797	0.5483	0.3886	0.006364 **	0.2846	0.1325	0.2279	0.09887	0.01654 *	0.8479	0.1744	0.361	0.1451	0.2824	0.1716	0.3776	0.5248	0.3334
BQ5, two groups: x<7 (n_1=274), x>=7 (n_2=399)	0.7408	0.9772	0.1047	0.292	0.4035	0.002381 **	0.1343	0.05747	0.00434 **	0.003607 **	0.01683 *	0.8051	0.6267	0.1929	0.1205	0.0271 *	0.03034 *	0.08549	0.06951	0.5023
BQ5, three groups: x<6 (n_1=190), 6<=x<8 (n_2=271), x>=8 (n_3=212)	0.1532	0.2886	0.07562	0.3356	0.06069	0.00926 **	0.1414	0.01166 *	0.0006896 ***	0.0005286 ***	0.168	0.6902	0.2367	0.2365	0.09009	0.03014 *	0.03286 *	0.09426	0.3523	0.01388 *
BQ6, two groups: x<7 (n_1=318), x>=7 (n_2=355)	0.9496	0.4908	0.5658	0.7009	0.5836	0.005581 **	0.3036	0.1441	0.1953	0.289	0.0005744 ***	0.5883	0.9916	0.9809	0.9041	0.4146	0.4478	0.6883	0.05302	0.9842
BQ6, three groups: x<6 (n_1=240), 6<=x<8 (n_2=229), x>=8 (n_3=204)	0.6204	0.9745	0.7312	0.8557	0.44	0.01988 *	0.3726	0.1359	0.1467	0.1851	0.009845 **	0.9204	0.6588	0.6927	0.602	0.6682	0.7609	0.625	0.262	0.6617
BQ7, two groups: x<7 (n_1=201), x>=7 (n_2=472)	0.1691	0.2554	0.8877	0.07533	0.6983	0.000459 ***	0.7582	0.6412	0.3008	0.3424	0.00778 **	0.1283	0.3437	0.1559	0.5785	0.7799	0.9402	0.8928	0.004751 **	0.5095
BQ7, three groups: x<6 (n_1=143), 6<=x<8 (n_2=214), x>=8 (n_3=316)	0.8834	0.7608	0.5168	0.3388	0.8597	0.004156 **	1	0.6272	0.4356	0.6223	0.003545 **	0.349	0.6863	0.687	0.948	0.9213	0.9675	0.7298	0.1105	0.684
BQ8, two groups: x<2 (n_1=123), x>=2 (n_2=550)	0.5048	0.2059	0.003137 **	0.0001024 ***	0.8198	0.5797	0.09938	0.02251 *	0.01417 *	0.00504 **	0.005765 **	0.0001656 ***	0.02226 *	1.453e-08 ***	1.59e-08 ***	0.01594 *	0.03187 *	0.02422 *	0.2095	0.7081
BQ9, two groups: x<51 (n_1=333), x>=51 (n_2=340)	6.116e-09 ***	3.613e-09 ***	4.392e-06 ***	0.000614 ***	1.18e-06 ***	0.07347	0.01332 *	0.04664 *	0.1505	0.0585	0.04851 *	0.742	0.02965 *	9.661e-05 ***	9.863e-05 ***	0.9912	0.978	0.8095	0.01925 *	0.3238
BQ9, three groups: x<40 (n_1=225), 40<=x<60 (n_2=231), x>=60 (n_3=217)	1.808e-07 ***	2.82e-08 ***	6.349e-06 ***	0.0004377 ***	3.191e-05 ***	0.2526	0.004295 **	0.01155 *	0.2856	0.0245 *	0.001738 **	0.2289	0.222	0.0002202 ***	0.000565 ***	0.8223	0.8839	0.8217	0.03513 *	0.02525 *

Supplementary Table 6. Results of one-way analysis of variance (ANOVA) between two groups and between three groups to identify statistically significant rating differences for expression statements ES1-ES20 in respect to groupings based on the answer values of each background question (BQ).
This table shows the p-values concerning the rating differences between groups when the statistical significance levels were defined as p<0.05, p<0.01 and p<0.001, denoted by symbols *, ** and ***, respectively (n=673).

Grouping based on the answer value of the background question	*ES1*	*ES2*	*ES3*	*ES4*	*ES5*	*ES6*	*ES7*	*ES8*	*ES9*	*ES10*	*ES11*	*ES12*	*ES13*	*ES14*	*ES15*	*ES16*	*ES17*	*ES18*	*ES19*	*ES20*
BQ1, two groups: x<7 (n_1=263), x>=7 (n_2=410)	0.833	0.814	0.253	0.761	0.184	0.000523 ***	0.031 *	0.00591 **	0.00703 **	0.00759 **	0.0427 *	0.257	0.579	0.512	0.158	0.0348 *	0.0101 *	0.0322 *	0.231	0.87
BQ1, three groups: x<6 (n_1= 218), 6<=x<8 (n_2=207), x>=8 (n_3=248)	0.646	0.23	0.561	0.705	0.0945	0.00115 **	0.186	0.0325 *	0.0368 *	0.0561	0.00935 **	0.845	0.711	0.8	0.469	0.375	0.115	0.16	0.118	0.474
BQ2, two groups: x<2 (n_1=219), x>=2 (n_2=454)	0.483	0.211	0.0237 *	0.405	0.653	0.00207 **	0.247	0.0905	0.0135 *	0.0174 *	0.00104 **	0.727	0.414	0.0724	0.0194 *	0.0733	0.0461 *	0.0801	0.452	0.0966
BQ4, two groups: x<2 (n_1=364), x>=2 (n_2=309)	0.548	0.502	0.543	0.535	0.748	0.0037 **	0.299	0.0653	0.185	0.0661	0.0176 *	0.849	0.177	0.315	0.093	0.249	0.135	0.341	0.456	0.453
BQ5, two groups: x<7 (n_1=274), x>=7 (n_2=399)	0.448	0.811	0.181	0.765	0.467	0.00174 **	0.108	0.037 *	0.0108 *	0.00916 **	0.0123 *	0.707	0.592	0.279	0.137	0.0265 *	0.0232 *	0.0675	0.0545	0.775
BQ5, three groups: x<6 (n_1=190), 6<=x<8 (n_2=271), x>=8 (n_3=212)	0.432	0.526	0.0788	0.702	0.133	0.00783 **	0.105	0.00669 **	0.00069 ***	0.00109 **	0.125	0.624	0.217	0.397	0.0944	0.0211 *	0.0183 *	0.0726	0.303	0.169
BQ6, two groups: x<7 (n_1=318), x>=7 (n_2=355)	0.823	0.626	0.713	0.301	0.814	0.00346 **	0.254	0.112	0.189	0.424	0.000383 ***	0.703	0.953	0.821	0.877	0.443	0.428	0.684	0.0371 *	0.426
BQ6, three groups: x<6 (n_1=240), 6<=x<8 (n_2=229), x>=8 (n_3=204)	0.572	0.976	0.819	0.455	0.588	0.0119 *	0.316	0.108	0.159	0.288	0.00789 **	0.891	0.653	0.936	0.642	0.658	0.647	0.627	0.193	0.786
BQ7, two groups: x<7 (n_1=201), x>=7 (n_2=472)	0.179	0.239	0.654	0.00977 **	0.729	0.000157 ***	0.841	0.667	0.382	0.511	0.00576 **	0.137	0.363	0.092	0.65	0.79	0.908	0.921	0.00306 **	0.996
BQ7, three groups: x<6 (n_1=143), 6<=x<8 (n_2=214), x>=8 (n_3=316)	0.902	0.896	0.673	0.0935	0.783	0.00171 **	0.993	0.641	0.505	0.757	0.00286 **	0.377	0.709	0.444	0.777	0.959	0.984	0.745	0.0905	0.788
BQ8, two groups: x<2 (n_1=123), x>=2 (n_2=550)	0.86	0.4	0.0026 **	0.000519 ***	0.936	0.593	0.0924	0.0159 *	0.00782 **	0.00413 **	0.00849 **	0.00012 ***	0.0213 *	1e-07 ***	2.82e-07 ***	0.00918 **	0.0123 *	0.0162 *	0.242	0.919
BQ9, two groups: x<51 (n_1=333), x>=51 (n_2=340)	1.49e-07 ***	2.65e-08 ***	5.44e-07 ***	4.5e-05 ***	3.52e-05 ***	0.124	0.0149 *	0.00118 **	0.0157 *	0.00207 **	0.0479 *	0.568	0.0268 *	2.66e-06 ***	2.14e-06 ***	0.451	0.275	0.741	0.0375 *	0.11
BQ9, three groups: x<40 (n_1=225), 40<=x<60 (n_2=231), x>=60 (n_3=217)	3.51e-06 ***	2.3e-07 ***	4.82e-07 ***	2.2e-05 ***	0.000736 ***	0.313	0.00341 **	0.000308 ***	0.0263 *	0.000401 ***	0.00145 **	0.309	0.19	4.26e-06 ***	5.23e-06 ***	0.802	0.511	0.868	0.0672	0.00704 **

Supplementary Table 7. Guidance texts of the online questionnaire.

Gathering the "need for help" ratings for expression statements with an online questionnaire is illustrated in the following Supplementary Figure 1.

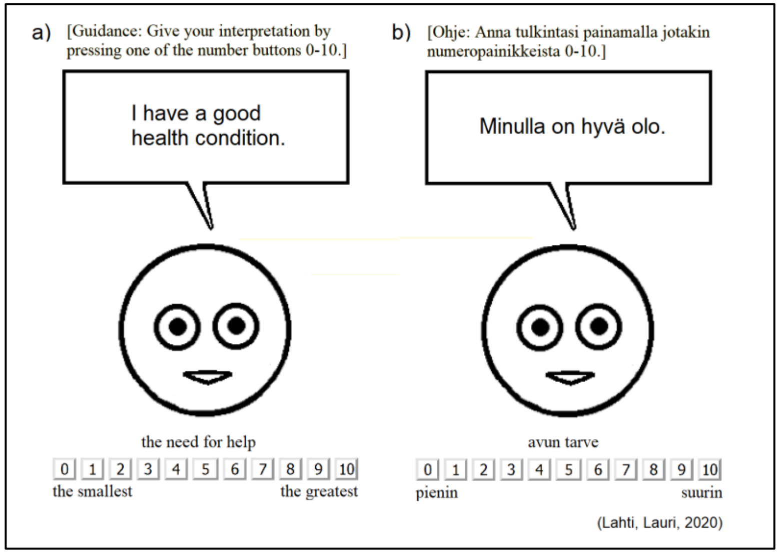

Supplementary Figure 1. Gathering the "need for help" rating for an expression statement on an 11-point Likert scale with an online questionnaire, shown here translated from Finnish to English (a) and in original formulation in Finnish (b).

Before the online questionnaire started to collect actual answers, the person was provided with the following guidance texts about how he/she should perform the interpretation tasks: "We ask you to evaluate different expressions, for example the expression 'I am happy'. Interpret how much each expression tells about the need for help. Give your interpretation about the expression on a numeric scale 0-10. 0 indicates the smallest possible need for help and 10 indicates the greatest possible need for help."

In Finnish: "Pyydämme sinua arvioimaan erilaisia ilmaisuja, esimerkiksi ilmaisua 'olen iloinen'. Tulkitse, kuinka paljon kukin ilmaisu kertoo avun tarpeesta. Anna tulkintasi ilmaisusta numeroasteikolla 0-10. 0 tarkoittaa mahdollisimman pientä avun tarvetta ja 10 tarkoittaa mahdollisimman suurta avun tarvetta."

Then a small training phase allowed the person to get accustomed to give the "need for help" ratings by rating three expression statements: "I have a good health condition.", "I have a bad health condition." and "I have an ordinary health condition."

In Finnish: "Minulla on hyvä olo.", "Minulla on huono olo." and "Minulla on tavallinen olo."

The answers that the person gave during the training phase were excluded from the data set that we use in the analysis reported in this our current research article.
After the training phase, the person was provided with the following guidance texts to still further clarify how he/she should perform the interpretation tasks: "Do not interpret how much the expression tells about just your own situation. Instead, interpret what kind of impression this expression induces in you. Thus give your interpretation about the expression's meaning in respect to the mentioned property."

In Finnish: "Älä tulkitse, kuinka paljon ilmaisu kertoo juuri sinun omasta tilanteestasi. Sen sijaan tulkitse, minkälaisen vaikutelman tämä ilmaisu herättää sinussa. Siis anna tulkintasi ilmaisun merkityksestä suhteessa mainittuun ominaisuuteen."

After showing those guidance texts, the person was allowed to start giving actual questionnaire answers, i.e. to perform the actual interpretation tasks.

Supplementary Table 8. Expression statements (ES) concerning the coronavirus COVID-19 epidemic that were rated by the person in respect to the impression about the "need for help".

Compact notation	*Expression statement*	*Expression statement in Finnish*	*Range of values for the person's answer (indicating the "need for help" rating)*
ES1	"I have a flu."	"Minulla on nuhaa."	0-10
ES2	"I have a cough."	"Minulla on yskää."	0-10
ES3	"I have a shortness of breath."	"Minulla on hengenahdistusta."	0-10
ES4	"My health condition is weakening."	"Yleistilani heikkenee."	0-10
ES5	"I have a sore throat."	"Minulla on kurkkukipua. "	0-10
ES6	"I have muscular ache."	"Minulla on lihassärkyä."	0-10
ES7	"I have a fever."	"Minulla on kuumetta."	0-10
ES8	"A sudden fever rises for me with 38 degrees of Celsius or more."	"Minulle nousee äkillinen kuume, joka on 38 astetta Celsiusta tai enemmän."	0-10
ES9	"I suspect that I have now become infected by the coronavirus."	"Epäilen, että olen nyt sairastunut koronavirukseen."	0-10
ES10	"I have now become infected by the coronavirus."	"Olen nyt sairastunut koronavirukseen."	0-10
ES11	"I am quarantined from meeting other people ordinarily so that the spreading of an infectious disease could be prevented."	"Olen eristettynä ihmisten tavanomaiselta tapaamiselta, jotta tartuntataudin leviäminen estyisi."	0-10
ES12	"I must be inside a house without getting out."	"Joudun olemaan talon sisällä ilman ulospääsyä."	0-10
ES13	"I must be without a human companion."	"Joudun olemaan ilman ihmisseuraa."	0-10
ES14	"I do not cope in everyday life independently without getting help from other persons."	"En pärjää arkielämässä itsenäisesti ilman avun saamista muilta henkilöiltä."	0-10
ES15	"I do not cope at home independently without getting help from persons who originate outside of my home."	"En pärjää kotona itsenäisesti ilman avun saamista kotini ulkopuolisilta henkilöiltä."	0-10
ES16	"I have an infectious disease."	"Minulla on tartuntatauti."	0-10
ES17	"I have an infectious disease that has been verified by a doctor."	"Minulla on tartuntatauti, jonka lääkäri on varmistanut."	0-10
ES18	"I suspect that I have an infectious disease."	"Epäilen, että minulla on tartuntatauti."	0-10
ES19	"I have a bad health condition."	"Minulla on huono olo."	0-10
ES20	"I have an ordinary health condition."	"Minulla on tavallinen olo."	0-10

Supplementary Table 9. Background questions (BQ) presented to the person.

Compact notation	*Question about the person's background information*	*Question about the person's background information in Finnish*	*Range of values for the person's answer*	*Range of values for the person's answer in Finnish*
BQ1: an estimated health condition	"What kind of health condition you have currently according to your opinion?" (de Bruin et al., 1996; Koskinen et al., 2012)	"Minkälainen terveydentilasi on mielestäsi nykyisin?" (de Bruin et al., 1996; Koskinen et al., 2012)	A 9-point Likert scale supplied with the following partial labeling: "9 Good", "8 –", "7 Rather good", "6 –", "5 Medium", "4 –", "3 Rather bad", "2 –", "1 Bad".	A 9-point Likert scale supplied with the following partial labeling: "9 Hyvä", "8 –", "7 Melko hyvä", "6 –", "5 Keskitasoinen", "4 –", "3 Melko huono", "2 –", "1 Huono".
BQ2: a health problem reduces ability	"Do you have a permanent or long-lasting disease or such deficit, ailment or disability that reduces your ability to work or to perform your daily living activities? Here the question refers to all long-lasting diseases identified by a doctor, and also to such ailments not identified by a doctor which have lasted at least three months but which affect your ability to perform your daily living activities." (Koskinen et al., 2012)	"Onko sinulla jokin pysyvä tai pitkäaikainen sairaus tai jokin sellainen vika, vaiva tai vamma, joka vähentää työ- tai toimintakykyäsi? Tässä tarkoitetaan kaikkia lääkärin toteamia pitkäaikaisia sairauksia sekä myös vähintään kolme kuukautta kestäneitä vaivoja, joita lääkäri ei ole todennut, mutta jotka vaikuttavat toimintakykyysi." (Koskinen et al., 2012)	No or yes	Ei or kyllä.
BQ3: one or more diseases identified by a doctor	"Has there been a situation that a doctor has identified in you one or several of the following diseases?" (Koskinen et al., 2012)	"Onko lääkäri joskus todennut sinulla jonkin/joitakin seuraavista sairauksista?" (Koskinen et al., 2012)	The person answers by selecting one or more options from a list of diseases (Koskinen et al., 2012), see Data analysis supplement (Additional file 1). For some options there is a question "other, what?" and an adjacent text input box so that the person can write some additional information concerning that option.	Henkilö vastaa valitsemalla yhden tai useampia vaihtoehtoja sairauksia sisältävästä luettelosta (Koskinen et al., 2012), katso Data analysis supplement (Additional file 1). Joidenkin vaihtoehtojen kohdalla on kysymys "muu, mikä?" ja sen vieressä tekstin syöttämislaatikko, johon henkilö voi kirjoittaa täydentävää tietoa koskien kyseistä vaihtoehtoa.
BQ4: a continuous or repeated need for a doctor's care	"Do you need continuously or repeatedly care given by a doctor for a long-lasting disease, deficit or disability that you have just mentioned?" (Koskinen et al., 2012)	"Tarvitsetko jatkuvasti tai toistuvasti lääkärinhoitoa jonkin äsken mainitsemasi pitkäaikaisen sairauden, vian tai vamman takia?" (Koskinen et al., 2012)	No or yes	En or kyllä.
BQ5: the quality of life	"How would you rate your quality of life? Give your estimate based on the latest two weeks." (Nosikov & Gudex 2003; Aalto et al., 2013)	"Minkälaiseksi arvioit elämänlaatusi? Anna arviosi viimeisimpien kahden viikon ajalta." (Nosikov & Gudex 2003; Aalto et al., 2013)	A 9-point Likert scale supplied with the following partial labeling: "9 Very good", "8 –", "7 Good", "6 –", "5 Neither good nor bad", "4 –", "3 Bad", "2 –", "1 Very bad".	A 9-point Likert scale supplied with the following partial labeling: "9 Erittäin hyväksi", "8 –", "7 Hyväksi", "6 –", "5 Ei hyväksi eikä huonoksi", "4 –", "3 Huonoksi", "2 –", "1 Erittäin huonoksi".
BQ6: the satisfaction about health	"How satisfied are you with your health? Give your estimate based on the latest two weeks." (Nosikov & Gudex 2003; Aalto et al., 2013)	"Kuinka tyytyväinen olet terveyteesi? Anna arviosi viimeisimpien kahden viikon ajalta." (Nosikov & Gudex 2003; Aalto et al., 2013)	A 9-point Likert scale supplied with the following partial labeling: "9 Very satisfied", "8 –", "7 Satisfied", "6 –", "5 Neither satisfied nor dissatisfied", "4 –", "3 Dissatisfied", "2 –", "1 Very dissatisfied".	A 9-point Likert scale supplied with the following partial labeling: "9 Erittäin tyytyväinen", "8 –", "7 Tyytyväinen", "6 –", "5 Ei tyytyväinen eikä tyytymätön", "4 –", "3 Tyytymätön", "2 –", "1 Erittäin tyytymätön".
BQ7: the satisfaction about ability	"How satisfied are you with your ability to perform your daily living activities? Give your estimate based on the latest two weeks." (Nosikov & Gudex 2003; Aalto et al., 2013)	"Kuinka tyytyväinen olet kykyysi selviytyä päivittäisistä toiminnoistasi? Anna arviosi viimeisimpien kahden viikon ajalta." (Nosikov & Gudex 2003; Aalto et al., 2013)	A 9-point Likert scale supplied with the following partial labeling: "9 Very satisfied", "8 –", "7 Satisfied", "6 –", "5 Neither satisfied nor dissatisfied", "4 –", "3 Dissatisfied", "2 –", "1 Very dissatisfied".	A 9-point Likert scale supplied with the following partial labeling: "9 Erittäin tyytyväinen", "8 –", "7 Tyytyväinen", "6 –", "5 Ei tyytyväinen eikä tyytymätön", "4 –", "3 Tyytymätön", "2 –", "1 Erittäin tyytymätön".
BQ8: the sex	"Tell what is your sex. The answer alternatives are similar as in the earlier health surveys of Finnish Institute for Health and Welfare (THL) to maintain comparability with the earlier results." (Koskinen et al., 2012)	"Kerro sukupuolesi. Vastausvaihtoehdot ovat samankaltaiset kuin aiemmissa THL:n terveystutkimuksissa, jotta säilyisi vertailtavuus aiempiin tuloksiin." (Koskinen et al., 2012)	Man or woman	Mies or nainen.
BQ9: the age	"Tell what is your age." (Koskinen et al., 2012)	"Kerro ikäsi." (Koskinen et al., 2012)	Age in years selected from the following range: 16 years, 17 years, ..., 99 years, 100 years or more	Age in years selected from the following range: 16 vuotta, 17 vuotta, ..., 99 vuotta, 100 vuotta tai enemmän.

Supplementary Table 10. Background question BQ3.

Compact notation	*Question about the person's background information*	*Question about the person's background information in Finnish*	*Range of values for the person's answer*	*Range of values for the person's answer in Finnish*
BQ3: one or more diseases identified by a doctor	"Has there been a situation that a doctor has identified in you one or several of the following diseases?" (Koskinen et al. 2012)	"Onko lääkäri joskus todennut sinulla jonkin/joitakin seuraavista sairauksista?" (Koskinen et al. 2012)	The person answers by selecting one or more options from a list of diseases (Koskinen et al., 2012), see Data analysis supplement (Additional file 1). For some options there is a question "other, what?" and an adjacent text input box so that the person can write some additional information concerning that option.	Henkilö vastaa valitsemalla yhden tai useampia vaihtoehtoja sairauksia sisältävästä luettelosta (Koskinen et al., 2012), katso Data analysis supplement (Additional file 1). Joidenkin vaihtoehtojen kohdalla on kysymys "muu, mikä?" ja sen vieressä tekstin syöttämislaatikko, johon henkilö voi kirjoittaa täydentävää tietoa koskien kyseistä vaihtoehtoa.

The person was asked to indicate if a doctor had identified one or more diseases in him/her and to describe them (BQ3) (in a form adapted from Koskinen et al., 2012).

Questions about one or more diseases identified by a doctor (in a form adapted from Koskinen et al., 2012)	*Questions about one or more diseases identified by a doctor in Finnish (in a form adapted from Koskinen et al., 2012)*
Has there been a situation that a doctor has identified in you one or several of the following diseases?	Onko lääkäri joskus todennut sinulla jonkin/joitakin seuraavista sairauksista?
Select all diseases that belong to your response in the following way:	Valitse kaikki vastaukseesi kuuluvat sairaudet seuraavasti:
Click the square that is on the left side of the name of the disease and then a check mark emerges in it. You can remove the selection with a new click.	Napauta sairauden nimen vasemmalla puolella olevaa ruutua, jolloin siihen ilmestyy valintamerkki. Voit poistaa valinnan uudella napautuksella.
If needed, answer to the questions "other, what?" in the following way: Click the box that is besides the question and write your answer into it.	Tarvittaessa vastaa kysymyksiin "muu, mikä?" seuraavasti: Napauta kysymyksen vieressä näkyvää laatikkoa ja kirjoita siihen vastauksesi.
Finally, press the button "I save my response and continue forward".	Lopuksi paina painiketta "Tallennan vastaukseni ja jatkan eteenpäin".
LUNG DISEASES	KEUHKOSAIRAUDET
[_] 1. asthma	[_] 1. astma
[_] 2. chronic obstructive pulmonary disease (COPD)	[_] 2. keuhkoputkien ahtauma (COPD)
[_] 3. inflammation of the bronchi (chronic bronchitis, lung catarrh)	[_] 3. krooninen keuhkoputkentulehdus (krooninen bronkiitti, keuhkokatarri)
HEART AND CIRCULATORY DISEASES	SYDÄN- JA VERISUONISAIRAUDET
[_] 4. heart attack, i.e., myocardial infarction	[_] 4. sydänveritulppa eli sydäninfarkti
[_] 5. coronary artery disease (atherosclerosis, angina pectoris)	[_] 5. sepelvaltimotauti (sepelvaltimoiden ahtauma, angina pectoris)
[_] 6. congestive heart failure	[_] 6. sydämen vajaatoiminta
[_] 7. high blood pressure, hypertension	[_] 7. kohonnut verenpaine, verenpainetauti
[_] 8. stroke (intracranial hemorrhage, cerebral infarction)	[_] 8. aivohalvaus (aivoverenvuoto, aivoveritulppa)
JOINT AND BACK DISEASES	NIVEL- JA SELKÄSAIRAUDET
[_] 9. rheumatoid arthritis	[_] 9. nivelreuma
[_] 10. arthrosis (osteoarthritis)	[_] 10. nivelkuluma (nivelrikko)
10.a. If you answered to the question 10 yes, in which joints it has been identified?	10a. Jos vastasit kysymykseen 10 kyllä, missä nivelissä se on todettu?
You can select several answer alternatives.	Voit valita useamman vastausvaihtoehdon.
[_] 10.1. knee	[_] polvi
[_] 10.2. pelvis	[_] lonkka
[_] 10.3. hand	[_] käsi
[_] 10.4. spine	[_] ranka
[_] 10.5. other, what? [____________________]	[_] muu, mikä? [____________________]
[_] 11. back disease or other back deficit	[_] 11. selkäsairaus tai muu selkävika
[_] 12. neck disease or other neck deficit	[_] 12. niskasairaus tai muu niskavika
INJURIES	TAPATURMAT
[_] 13. permanent disability caused by an injury	[_] 13. tapaturman aiheuttama pysyvä vamma
13a. If you answered to the question 13 yes, a what kind of permanent disability is it?	13a. Jos vastasit kysymykseen 13 kyllä, minkälainen pysyvä vamma on kyseessä?
You can select several answer alternatives.	Voit valita useamman vastausvaihtoehdon.
[_] 13.1. face or jaw injury	[_] kasvo- tai leukavamma
[_] 13.2. some other head or brain injury	[_] jokin muu pää- tai aivovamma
[_] 13.3. visual impairment	[_] näkövamma
[_] 13.4. hearing impairment	[_] kuulovamma
[_] 13.5. trauma in an upper limb or limbs	[_] vamma yläraajassa/-raajoissa
[_] 13.6. pelvis fracture or its consequence	[_] lonkkamurtuma tai sen jälkitila
[_] 13.7. some other trauma in a lower limb or limbs	[_] jokin muu vamma alaraajassa/-raajoissa
[_] 13.8. trauma in the body or back	[_] vamma vartalossa tai selässä
[_] 13.9. lung injury	[_] keuhkovamma
[_] 13.10. some other injury, what? [____________________]	[_] jokin muu vamma, mikä? [____________________]
MENTAL HEALTH PROBLEMS	MIELENTERVEYDEN ONGELMAT
[_] 14. psychic or mental health-related disease	[_] 14. psyykkinen tai mielenterveyteen liittyvä sairaus
14a. If you answered to the question 14 yes, a what kind of disease is it?	14a. Jos vastasit kysymykseen 14 kyllä, minkälainen sairaus on kyseessä?
You can select several answer alternatives.	Voit valita useamman vastausvaihtoehdon.
[_] 14.1. psychosis	[_] psykoosi
[_] 14.2. depression	[_] masennus
[_] 14.3. anxiety	[_] ahdistus
[_] 14.4. substance abuse problem	[_] päihdeongelma
[_] 14.5. other, what? [____________________]	[_] muu, mikä? [____________________]
VISION AND HEARING DEFICITS	NÄKÖ- JA KUULOVIAT
[_] 15. cataract	[_] 15. silmien harmaakaihi

15a. If you answered to the question 15 yes, have you been in an eye surgery due to it?	15a. Jos vastasit kysymykseen 15 kyllä, oletko ollut sen takia silmäleikkauksessa?
[_] Yes I have.	[_] Kyllä olen.
[_] 16. glaucoma (ocular hypertension, glaucoma disease)	[_] 16. silmien glaukooma (silmänpainetauti, viherkaihi)
[_] 17. macular degeneration	[_] 17. silmänpohjan rappeuma
[_] 18. hearing deficit, hearing disability or disease that weakens hearing	[_] 18. kuulovika, kuulovamma tai kuuloa heikentävä sairaus
OTHER DISEASES	MUUT SAIRAUDET
[_] 19. diabetes (diabetes mellitus)	[_] 19. diabetes (sokeritauti)
[_] 20. cancer disease (malignant tumor)	[_] 20. syöpätauti (pahanlaatuinen kasvain)
[_] 21. Parkinson's disease	[_] 21. Parkinsonintauti
[_] 22. involuntary urination, leakage of urine or urinary incontinence	[_] 22. virtsan pidättämisen vaikeuksia, virtsan karkailua tai inkontinenssi
23. Do you have still some other long-lasting disease, deficit, ailment or disability that a doctor has identified in you?	23. Onko sinulla vielä jokin muu lääkärin toteama pitkäaikainen sairaus, vika, vaiva tai vamma?
[_] Yes I have.	[_] Kyllä on.
[_] 23a. If you answered to the question 23 yes, this other thing is what? [____________________]	[_] 23a. Jos vastasit kysymykseen 23 kyllä, mikä muu? [____________________]
Button "I save my response and continue forward"	Painike "Tallennan vastaukseni ja jatkan eteenpäin"

Supplementary Table 11. Animation of the face figure that is shown together with the expression statements in the online questionnaire.

The online questionnaire shows each expression statement as a speech bubble and below that an animation of a face figure is shown. The animation is based on 18 image frames that are illustrated in this table. The animation is defined to present the frames 1-18 with the speed of 10 frames per second. In the end, the last frame 18 remains permanently displayed, thus there is no looping of the animation.

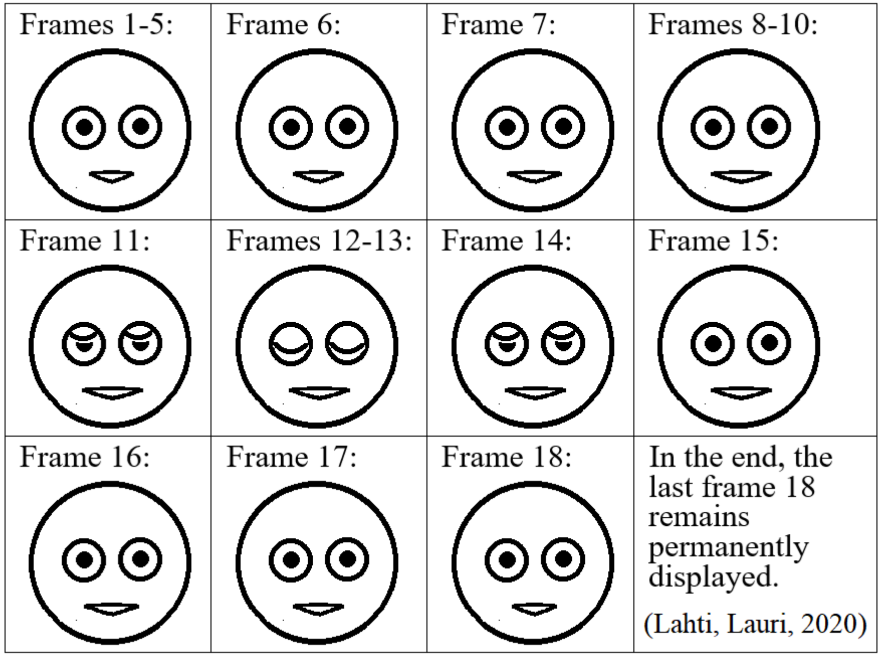

Supplement: Supplementary file 1 — Additional file 1. [file 12874_2021_1502_MOESM1_ESM.pdf]
